# A new type of convergent paired electrochemical synthesis of sulfonamides under green and catalyst-free conditions

**DOI:** 10.1038/s41598-023-44912-y

**Published:** 2023-10-16

**Authors:** Pouria Patoghi, Ali Sadatnabi, Davood Nematollahi

**Affiliations:** Faculty of Chemistry, Bu-Ali-Sina University, Hamedan, 65174-38683 Iran

**Keywords:** Electrochemistry, Reaction mechanisms

## Abstract

Our main goal in this work is to synthesize valuable sulfonamide compounds according to the principles of green chemistry and also to present a unique convergent paired mechanism for their synthesis. In this study, we introduced a new type of convergent paired electro-organic synthesis of sulfonamide derivatives via a catalyst, oxidant, halogen and amine-free method. In this research, instead of using toxic amine compounds, an innovative mechanism based on the reduction of nitro compounds and in-situ production of amine compounds was used. The mechanism of electrophile generation is the cathodic reduction of the nitro compound to the hydroxylamine compound and then the anodic oxidation of the hydroxylamine to the nitroso compound. On the other hand, the nucleophile generation mechanism involves the two-electron oxidation of sulfonyl hydrazide to related sulfinic acid at the anode surface. The reaction leading to the synthesis of sulfonamides involves a one-pot reaction of the generated nitroso compound with the produced sulfinic compound.

## Introduction

Sulfonamides are known as valuable organic compounds with great biological and pharmaceutical activities^1^. They are widely used to develop the synthesis of new drugs and other pharmaceutically active components in medicinal and agricultural chemistry. Sulfonamide drugs have clinical applications such as antibacterial, anti-diabetic, anti-tuberculosis, anti-fungal antimalarial, etc^2–8^. In addition, they are used as additives in organic dyes to promote stability and lubricity^9^. To date, researchers have adopted various methods for the synthesis of sulfonamides and these efforts are still ongoing to provide a green method. As a general procedure, the synthesis of sulfonamides involves the coupling between a sulfonyl compound (often arenesulfonyl hydrazide and sodium arenesulfinate) and an amine (mostly of the primary and secondary types)^10^. The synthetic methods reported so far require special conditions and catalysts such as (I) the utilization of transition metals such as copper^11,12^, (II) the use of strong oxidants such as hydrogen peroxide (H_2_O_2_) or *tert*-butyl hydroperoxide (TBHP)^13–16^ and finally (III) In the presence of halogens such as iodine^17^. These methods suffered from some disadvantages such as the use of metals and oxidants as toxic/expensive reagents, harsh handling conditions, and the production of toxic waste; which is not in accordance with green chemistry principles^18,19^. In contrast, in electro-organic synthesis, electrons are used as green and cost-effective reactant, which has advantages such as simplicity, minimizing the production of toxic waste and energy consumption, and being eco-friendly^10,20^. Consequently, the demand for using metal catalysts and hazardous oxidants is eliminated^21–24^.

The unique features mentioned above were related to conventional electrosynthesis reactions. In these methods, when the desired electrochemical reaction is performed on the working electrode, the opposite electrochemical reaction occurs simultaneously at the counter electrode. The reactions that are carried out on the counter electrode are usually reduction or oxidation of the solvent, and their task is to consume the electrons produced in the working electrode or to supply the necessary electrons for the working electrode process. Now, if by changing the counter electrode reaction, to the production of the product or helps to produce the final product, the conventional electrosynthesis process has become “*paired electrosynthesis*”^24,25^. This method saves energy and time. Since in this method one mole of electrons can perform useful reactions in the working and counter electrodes and since the total current efficiency is the sum of the reactions that occur in each electrode, the current efficiency can increase up to 200% when cathodic and anodic processes produce the same product. Useful and complete information about the "*paired electrosynthesis*" can be obtained from the references^24,25^.

**Figure 1:**
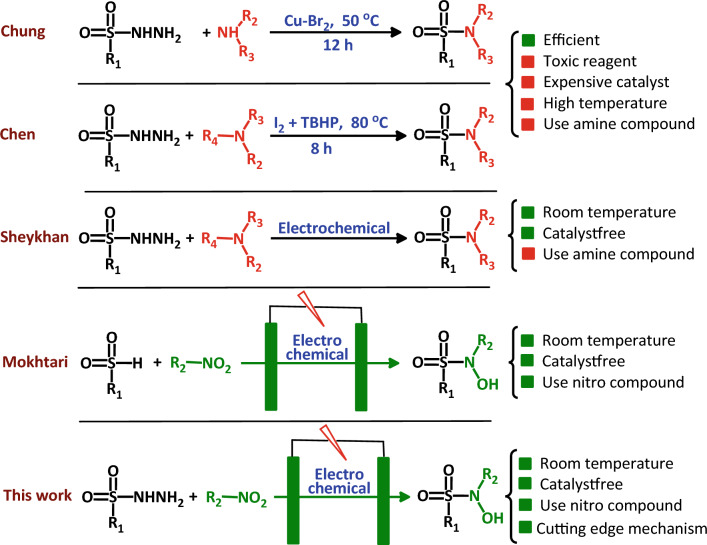
Schematic representation of synthesis methods of sulfonamide derivatives. The structures of the compounds were drawn using ChemOffice 12.0 (CambridgeSoft).

It should be noted that, in chemical synthesis, sulfnyl hydrazides are considered as an alternative to sulfinic acids because these compounds have advantages over other sulfonyl derivatives, including the fact that they are stable, non-corrosive, and moisture compatible^20^. Figure 1 compares the methods of synthesizing sulfonamides using sulfonyl hydrazides in recent years with the method proposed in this research.

The first two methods are very efficient chemical methods, but they have the disadvantage of using toxic reagents, expensive catalysts, high temperature and amine molecules^26,27^. Although the third method^28^, which is an electrochemical method, has solved most of the problems of the first two methods, it still has one problem: the use of amines which are toxic^29^ as a starting material. In the fourth method, sulfonyl hydrazide is not used and it seems that it should not be here, but because of its unique features and the lack of use of toxic amine, it is included here for comparison with the results of this research^30,31^. But, in this work, we presented a new strategy based on the activation of sulfonyl hydrazides and nitro compounds at the anode and the cathode without using any kind of catalyst or oxidant. It should be noted that so far no report has been published regarding the chemical or electrochemical reaction of nitro compounds and sulfonyl hydrazides.

In this method, by reducing the nitro compound to hydroxylamine on the cathode surface and then the oxidation of the formed hydroxylamine on the anode surface to the nitroso compound (electrophile formation process) on the one hand, and the oxidation of sulfonyl hydrazide to sulfonic acid on the anode surface (nucleophile formation process) on the other hand, the conditions for their reaction in water and the synthesis of sulfonamides are provided. In this method, the only by-product produced is nitrogen gas, which is a safe by-product. Replacing amines with nitro compounds minimizes the production of toxic waste products, which has created more favourable conditions in terms of green chemistry^32^.

## Experimental data

### Reagents and apparatus

All voltammetric experiments including cyclic voltammetry and controlled potential coulometry were carried out by a µ-Autolab model PGSTAT 20 potentiostat/galvanostat (Metrohm-Autolab, Netherland) and nova 1.10 software was used to control of the µ-Autolab instruments with USB connection and collect the data. For electrochemical studies, a three-electrode system in an undivided cell (all electrodes from AZAR Electrodes) consisting of a glassy carbon (GC) disc as a working electrode (2.8 mm diameter), platinum wire as the counter electrode, and Ag/AgCl (3 M) as the reference electrode was used. In macroscale electrolysis, cathode electrodes that are used were the assembly of four carbon rods with a total surface area of 32 cm^2^, while in the case of the anode, it consisted of a carbon rod (8 cm^2^ area). It should be noted that before each voltammetric experiment, the GC electrode was polished using alumina slurry and cleaned with acetone. Subsequently, it was completely rinsed with distilled water. Nitrobenzene (**NB1**), *p*-nitrotoluene (**NB2**), acetonitrile, phosphoric acid, *n*-hexane, and ethyl acetate were purchased from Sigma-Aldrich Company and were used as received, without further purification. Silica gel (SiO_2_, 60 Å silica gel, Merck Grade, 70–230 mesh) was obtained from Merck Company. *p*-Chloronitrobenzene (**NB3**) and *p*-Iodonitrobenzene (**NB4**) were synthesized according to previously described methods^33,34^. The FTIR spectra of products were recorded in the range 500–4000 cm^−1^ using a Perkin‐Elmer 1760. The ^1^H and ^13^C NMR spectra were recorded in CDCl_3_ on a Bruker Avance 400 MHz spectrometer. Mass spectrometry analysis was performed in electron impact mode at an ionization potential of 70 eV using an Agilent-5973 mass spectrometer. The melting point of the products was determined using a Barnstead Electrothermal 9100 instrument.

### Synthetic procedures for arenesulfonyl hydrazides

Benzenesulfononyl hydrazide (**SH1**) and *p*-Toluenesulfonyl hydrazide (**SH2**) were synthesized according to the previous published method^35^. Briefly, 10 mmol of 4-methyl benzenesulfonyl chloride or benzenesulfonyl chloride was poured into a 25 mL beaker. In the following, 20 mL of THF solvent was added to it and stirred for 5 min at room temperature (RT) until it completely dissolved. On the other hand, in a 50 mL bottom flask, 30 mmol of hydrazine was poured and placed at a temperature of 0–5 °C. The prepared solution was added drop by drop to the flask containing hydrazine and after that, the mixture was stirred for 30 min at a temperature of 0–5 °C. After this time, the mixture was stirred for another 1 h at room temperature. After the completion of the reaction, water and ethyl acetate were added to the reaction mixture, and after decanting, the organic layer was separated and sodium sulphate was added to it. In the last step, the organic layer was filtered and its solvent was evaporated. A pure white product was obtained (product yield is more than 95%).

### Electrochemical synthesis of sulfonamide derivatives

A mixture of arensulfonyl hydrazide (0.5 mmol) and nitroaren (0.5 mmol) was electrolyzed at − 0.6 V versus Ag/AgCl in an undivided cell containing a solution (ca. 80 mL) of water (phosphate buffer, pH = 2.0, *c* = 0.2 M)/acetonitrile (50/50 v/v) at room temperature (RT). The electrolysis was terminated when the decay of the current became more than 95%. At the end of electrolysis, after extracting the reaction solution with ethyl acetate, the organic layer was allowed to dry overnight. Purification was performed using thin layer chromatography (eluent 4:1 *n*-hexane/ethyl acetate) to afford the sulfonamide as the product. The final product was characterized by H-NMR, FTIR and mass spectroscopy (Supplementary Information). The structures of known sulfonamides were confirmed by the comparison of their spectroscopic data with those previously reported. It should be noted that acetonitrile has been used as a co-solvent for the complete dissolution of the required precursors.

### Characteristics of the products

*N-Hydroxy-4-methyl-N-phenylbenzenesulfonamide (****SU1****):* Pale yellow; MP = 137–139 °C (Lit. 139–141 °C)^30^; IR (KBr, cm^−1^): 3377 (medium, O–H), 2856 (weak, CH_3_), 1590 (medium C=C), 1447, 1347, 1172 (strong, S=O). ^1^H-NMR (400 MHz, DCl_3_) *δ* 7.40 (d, *J* = 8.3 Hz, 2H), 7.24 (d, *J* = 0.9 Hz, 2H), 7.22 (d, *J* = 3.1 Hz, 2H), 7.20 (s, 1H), 7.17–7.13 (m, 2H), 2.42 (s, 3H).

*N-Hydroxy-4-methyl-N-(p-tolyl)benzenesulfonamide (****SU2****):* Pale yellow; MP: 88–90 °C, IR (KBr, cm^−1^): 3360 (medium, O–H), 2922 (weak, CH_3_) 1596 (medium C=C), 1452, 1338 and 1162 (strong, S=O). MS (*m/z*) (EI, 70 eV) (relative intensity): 91.2 (95), 155.2 (75), 276.1 (100). ^1^H-NMR (400 MHz, CDCl_3_) *δ* 7.43 (d, *J* = 8.0 Hz, 2H), 7.22 (d, *J* = 7.9 Hz, 2H), 7.03 (q, *J* = 8.5 Hz, 4H), 2.42 (s, 3H), 2.32 (s, 3H).

*N-(4-Chlorophenyl)-N-hydroxy-4-methylbenzenesulfonamide (****SU3****):* Pale yellow; MP: 125–127 °C (Lit. 129–130 °C)^27^; IR (KBr, cm^−1^): 3347 (medium, O–H), 2923 (weak, CH_3_), 1595 (medium C=C), 1482, 1343 and 1163 (strong, S=O). ^1^H-NMR (400 MHz, CDCl_3_) *δ* 7.42 (d, *J* = 8.1 Hz, 2H), 7.24 (d, *J* = 2.9 Hz, 2H), 7.22 (d, *J* = 3.7 Hz, 2H), 7.10 (d, *J* = 8.8 Hz, 2H), 2.42 (s, 3H).

*N-Hydroxy-N-phenylbenzenesulfonamide (****SU4****):* MP = 129–131 °C (Lit. 133–134 °C)^30^; IR (KBr, cm^−1^): 3376 (medium, O–H), 1590 (medium C=C), 1486, 1347, 1172 (strong, S=O). ^1^H-NMR (400 MHz, CDCl_3_) *δ* 7.62 (t, *J* = 7.4 Hz, 1H), 7.54 (d, *J* = 7.6 Hz, 2H), 7.42 (t, *J* = 7.7 Hz, 2H), 7.25 (d, *J* = 6.6 Hz, 2H), 7.17–7.12 (m, 3H).

*N-(4-Chlorophenyl)-N-hydroxybenzenesulfonamide (****SU5****):* Pale yellow; MP: 101–103 °C (Lit. 102–104 °C)^27^; IR (KBr, cm^−1^): 3370 (medium, O–H), 1578 (medium C=C), 1484, 1347 and 1170 (strong, S=O). ^1^H-NMR (400 MHz, CDCl_3_) *δ* 7.56–7.48 (m, 1H), 7.43 (d,* J* = 7.4 Hz, 2H), 7.37–7.29 (m, 2H), 7.11 (d, *J* = 8.3 Hz, 2H), 6.98 (d, *J* = 8.3 Hz, 2H).

*N-Hydroxy-N-(4-iodophenyl)benzenesulfonamide (****SU6****):* Pale yellow; MP: 106–109 °C (Lit. 108–110 °C)^27^; IR (KBr, cm^−1^): 3339 (medium, O–H), 1446 (medium C=C), 1340 and 1166 (strong, S=O). ^1^H-NMR (400 MHz, CDCl_3_) *δ* 7.63 (d, *J* = 7.4 Hz, 2H), 7.55 (d, *J* = 9.1 Hz, 2H), 7.48–7.41 (m, 3H), 6.90 (d, *J* = 8.6 Hz, 2H).

## Results and discussion

**Figure 2:**
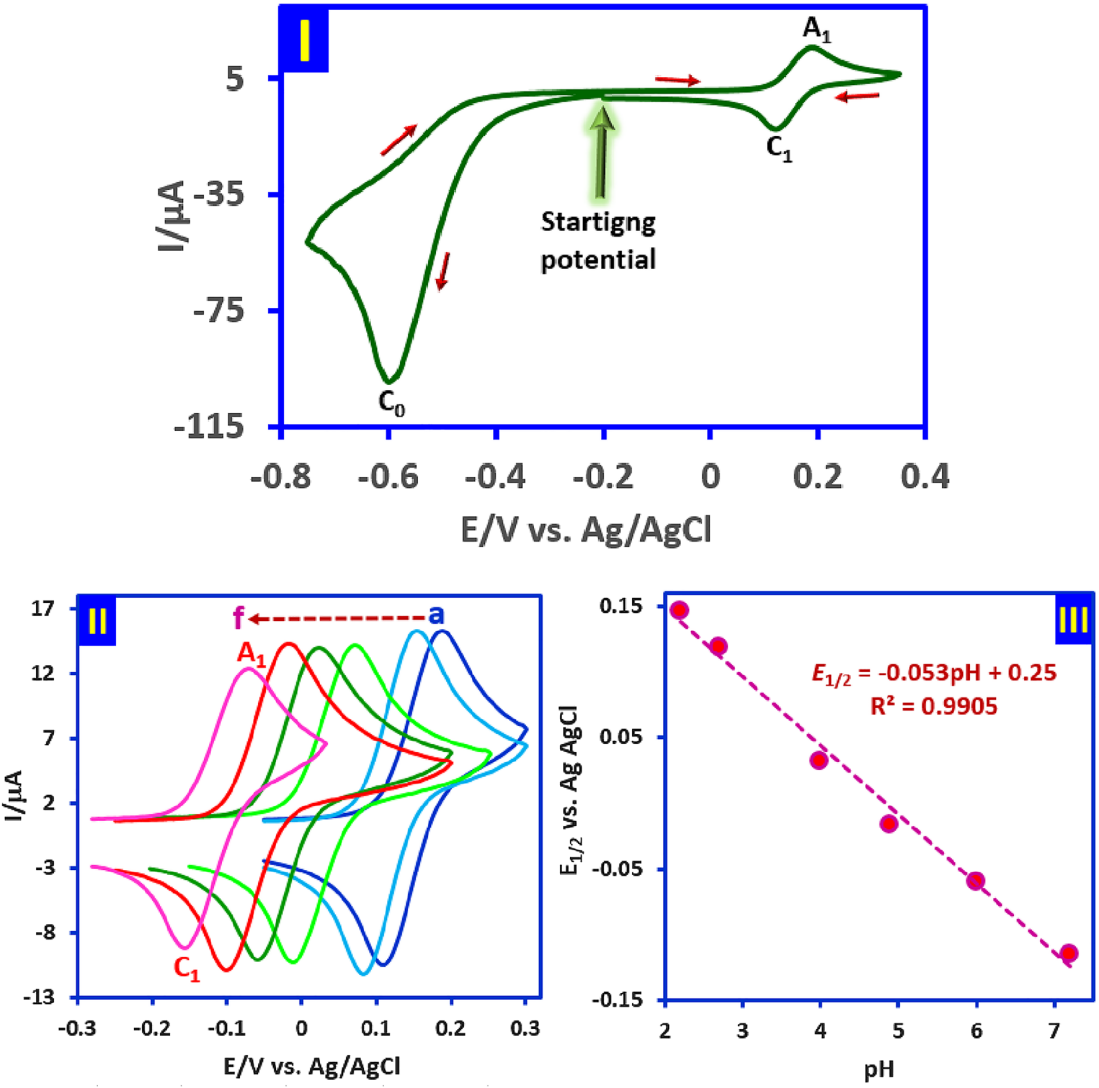
Part I: Cyclic voltammogram of 1.0 mM **NB2** in aqueous phosphate buffer (pH, 2.0, *c* = 0.2 M)/acetonitrile (50/50 v/v). Scan rate: 100 mV/s at room temperature. Part II: Cyclic voltammograms of **HA2/NS2** redox couple (1.0 mM) in aqueous buffer/acetonitrile (50/50, v/v) mixture with different pH values and same ion strength at scan rate of 100 mV s^−1^. The pH of buffer solutions from (**a**) to (**f**) are 2.2, 2.7, 4.0, 4.9, 6.0 and 7.2, respectively. Part III: Pourbaix diagram of **HA2/NS2** redox couple. Working electrode: Glassy carbon electrode. All experiments are performed at room temperature. These figures were prepared by Microsoft Excel (OFFICE 2013).

**Figure 3:**
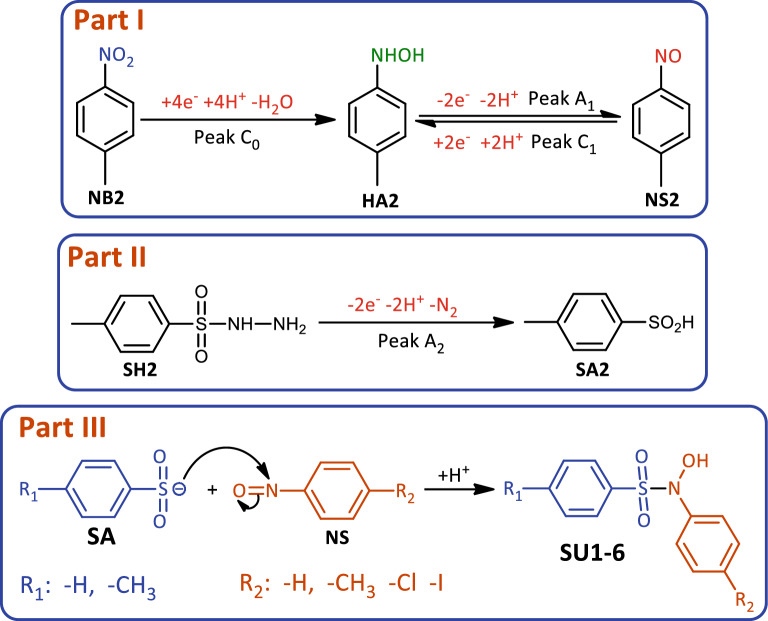
Part I: The redox behavior observed in Fig. 2. Part II: The oxidation mechanism of **SH2**. Part III: The reaction of produced nucleophile with the generated electrophile.

To study the electrochemical behavior of *p*-nitrotoluene (**NB2**), cyclic voltammogram (CV) of **NB2** at a glassy carbon electrode, in aqueous phosphate buffer (pH = 2.0, *c* = 0.2 M)/acetonitrile (50/50 v/v) at scan rate of 100 mV s^−1^ is shown in Fig. 2, part I. When the cathodic scan is performed first, the voltammogram consists of an irreversible well defined cathodic peak (C_0_) at − 0.60 V versus Ag/AgCl attributed to a four-electron reduction of the nitro group of **NB2** to hydroxylamine group in the forward scan, and a reversible couple (A_1_/C_1_) at 0.19 and 0.12 V versus Ag/AgCl, respectively, in the reverse scan. The anodic peak of A_1_ corresponds to the oxidation of cathodically generated *N*-(*p*-tolyl)hydroxylamine (**HA2**) to related nitroso compound, *p*-nitrosotoluene (**NS2**) and its cathodic counterpart (C_1_) which is related to the reduction of **NS2** to **HA2** (Fig. 3)^30,31^. It should be noted that the existence of peaks A_1_ and C_1_ strongly depends on the direction of the potential scan. So when the potential is initially scanned from 0.0 to 0.5 V versus Ag/AgCl, none of the peaks A_1_ and C_1_ are observed.

In the next part of this work, the effect of pH on the **HA2**/**NS2** redox couple was investigated in detail by cyclic voltammetry (Fig. 2, part II). The results showed that *E*_1/2_ (half-wave potential) of **HA2**/**NS2** redox couple is pH-dependent and changes to less positive potentials with increasing pH. This shift confirms the presence of the proton(s) in the **HA2**/**NS2** redox process as shown in Fig. 3, part I. Based on this, the Pourbaix diagram related to the **HA2**/**NS2** redox couple has been reported in the range of pH 2 to 7 (Fig. 2, part III). In this pH range, the plot shows a straight line with a slope of 0.053 mV/pH, which corresponds to the theoretical value (0.059 mV/pH) for the two-electron/two-proton process.

**Figure 4:**
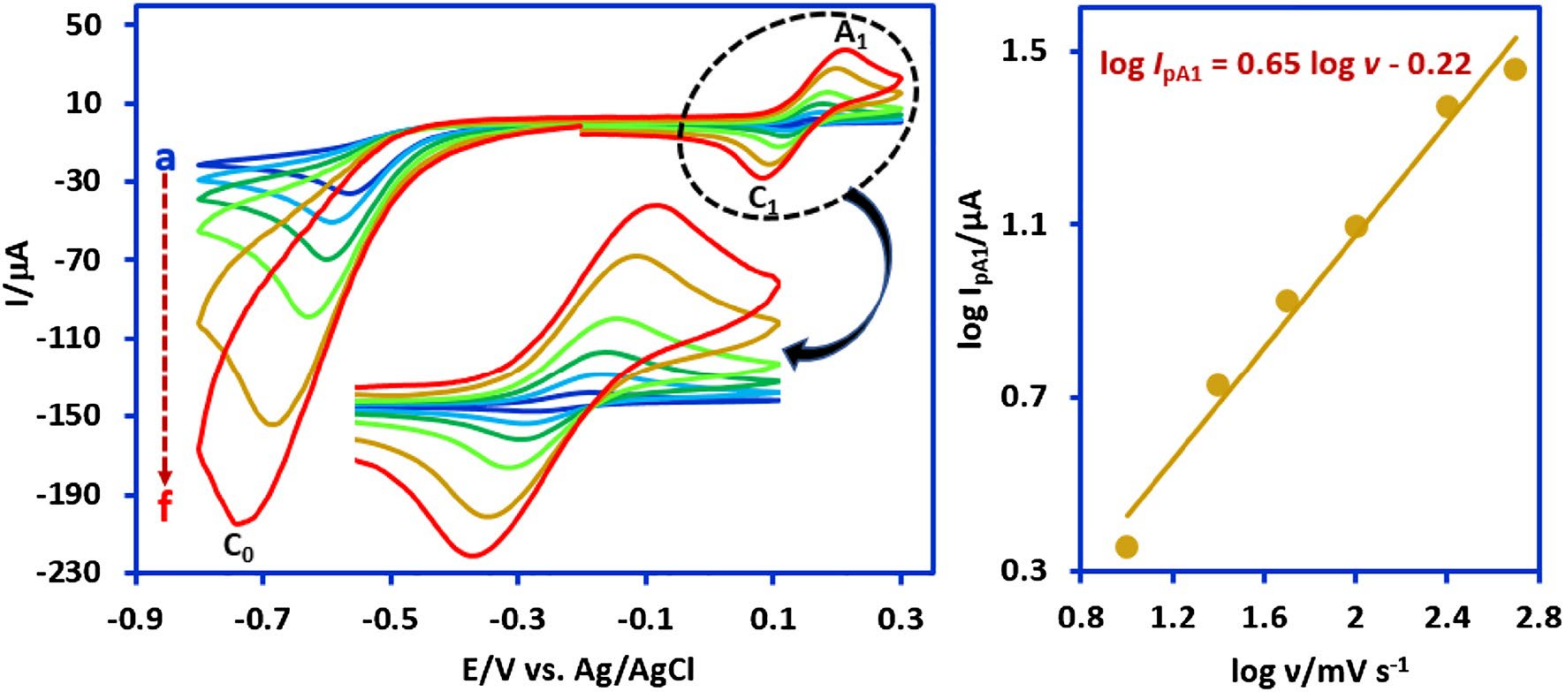
Left: The effect of potential scan rate on the cyclic voltammograms **NB2** (1.0 mM) in water (phosphate buffer, pH, 2.0, *c* = 0.2 M)/acetonitrile (50/50, v/v) mixture. Scan rates from (**a**) to (**f**) are: 10, 25, 50, 100, 250 and 500 mV s^−1^. Right: The plot of log *I*_pA1_ versus log *ν* at glassy carbon electrode at room temperature.

To obtain more details on the electrochemical properties of **HA2** produced by **NB2** reduction, the effect of the potential scan rate on the cyclic voltammogram of **NB2** has been investigated (Fig. 4). The findings show that the peak current ratio (*I*_pC1_/*I*_pA1_) depends on the scan rate. This ratio increases with increasing scan rate, which indicates the occurrence of a chemical reaction after the electron transfer step. In addition, adsorption-diffusion behavior of **HA2** was investigated. To achieve this goal, the log *I*_pA1_ was plotted against log scan rate (log *v*) at pH = 2.0. The results lead to a straight line with a slope of 0.65 (Fig. 4). This slope is lower than the theoretical value of 1 for the adsorption-controlled process and higher than the theoretical value of 0.5 for the diffusion-controlled process. Accordingly, adsorption-diffusion behavior has been proposed for **HA2** oxidation process at a glassy carbon electrode in aqueous phosphate buffer (pH, 2.0, *c* = 0.2 M)/acetonitrile (50/50 v/v) mixture. These results show that the oxidation process of hydroxylamine (**HA**) on the anode surface is not purely adsorptive and therefore the amount of **HA** on the anode surface during the electrolysis depends on various parameters such as the solution stirring speed, anode–cathode distance, solution viscosity, etc.

**Figure 5:**
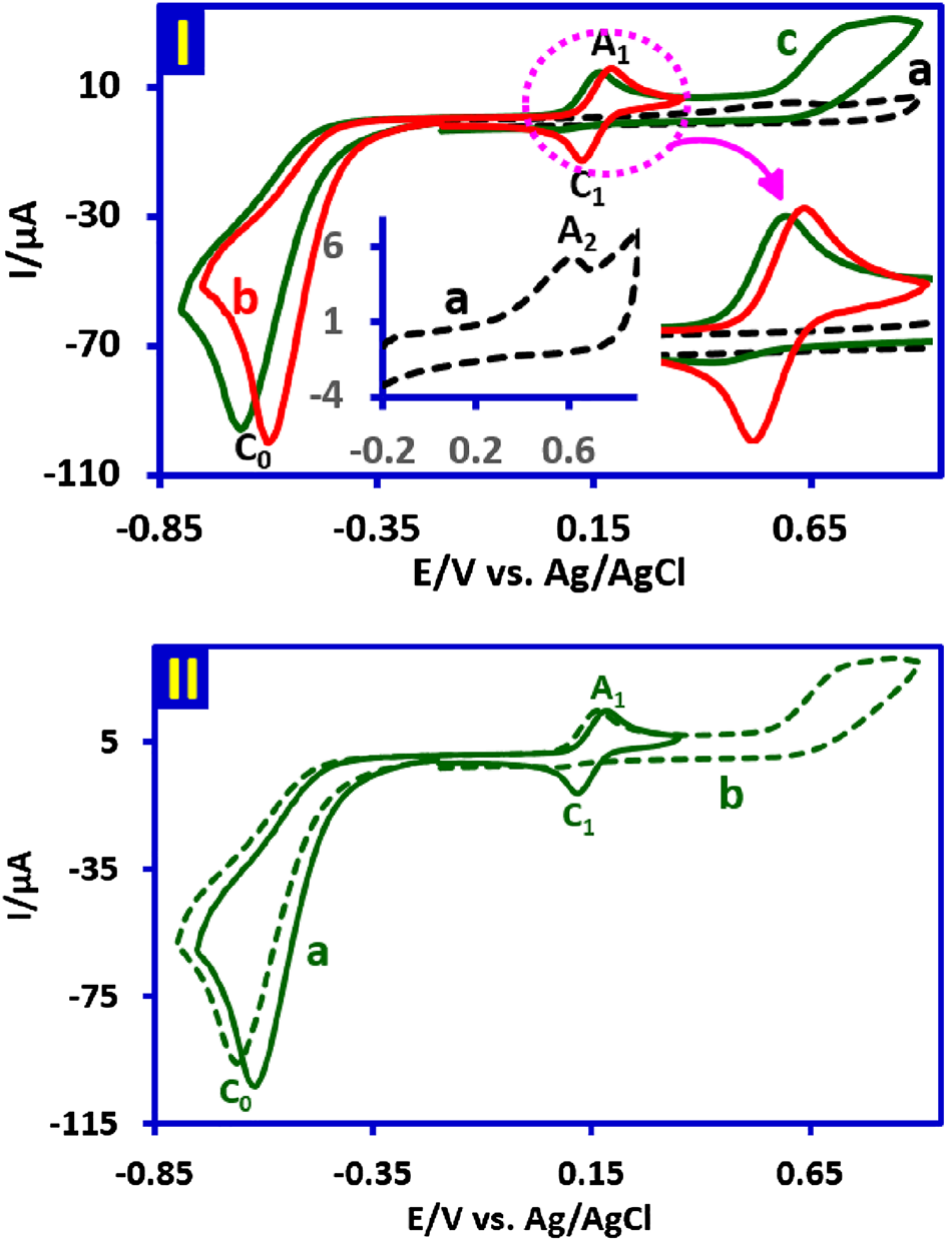
Part I: (**a** and inset) Cyclic voltammogram of **SH2** (1.0 mM). (**b**) Cyclic voltammogram of 1.0 mM of **NB2** in the absence of **SH2**. (**c**) Cyclic voltammogram of 1.0 mM of **NB2** in the presence of **SH2** (1.0 mM). Part II: Cyclic voltammograms of 1.0 mM of **NB2** in the presence of **SH2** (1.0 mM) (**a**) Anodic switching potential + 0.35 V versus Ag/AgCl. (**b**) Anodic switching potential + 0.90 V versus Ag/AgCl. Solvent: water (phosphate buffer, pH, 2.0, *c* = 0.2 M) /acetonitrile (50/50, v/v) mixture. Scan rate: 100 mV s^−1^. Working electrode: Glassy carbon electrode. All experiments are performed at room temperature. These figures were prepared by Microsoft Excel (OFFICE 2013).

Cyclic voltammogram of *p*-toluenesulfonyl hydrazide (**SH2**) was shown in Fig. 5, part I, curve a and inset. **SH2** has an irreversible anodic peak at 0.62 V versus Ag/AgCl, attributed to two-electron oxidation of **SH2** to *p*-toluenesulfinic acid (**SA2**) according to Fig. 3, part II^36^. In the next steps, we use this compound (**SA2**) as a nucleophile. In almost all electrochemical syntheses, nucleophiles are generated at the cathode by electron injection from cathode to a molecule. This study is one of the few studies in which the generation of nucleophile has been performed at the anode by taking electrons from the molecule. In this Figure, curve b is the cyclic voltammogram of **NB2**, which was recorded under the same conditions as Fig. 2. Unlike individual voltammograms a and b, under similar conditions, the cyclic voltammogram of **NB2** in the presence of **SH2** is shown in curve c. This voltammogram is recorded in such a way that **SH2** is also oxidized in the anodic scan (switching anodic potential more than *E*_pA2_). The effect of oxidized form of **SH2** (**SA2**) on the cyclic voltammogram of **NB2** is shown in Fig. 5, part I, curve c. The disappearance of the cathodic peak C_1_ confirms the chemical reaction between **SA2** and **NS2**.

To confirm the necessity of oxidation of **SH2** to create an active nucleophile (**SA2**) for reaction with **NS2**, in another experiment the anodic scan of **NB2** in the presence of **SH2** was limited to + 0.35 V (before *E*_pA2_). The cyclic voltammogram of **NB2** in the presence of **SH2** with a truncated anodic switching potential (ASP) is shown in Fig. 5, part II, curve a and is compared with the cyclic voltammogram of **NB2** in the presence of **SH2** with a higher anodic scan range (*E*_ASP_ = 0.90 V) (curve b). The comparison of two voltammograms (a and b) clearly shows that the removal of peak C_1_ occurs when the anodic switching potential becomes more positive than the *E*_pA2_. In other words, the presence of **SA2** on the surface of the electrode is necessary for the reaction with **NS2**.

**Figure 6:**
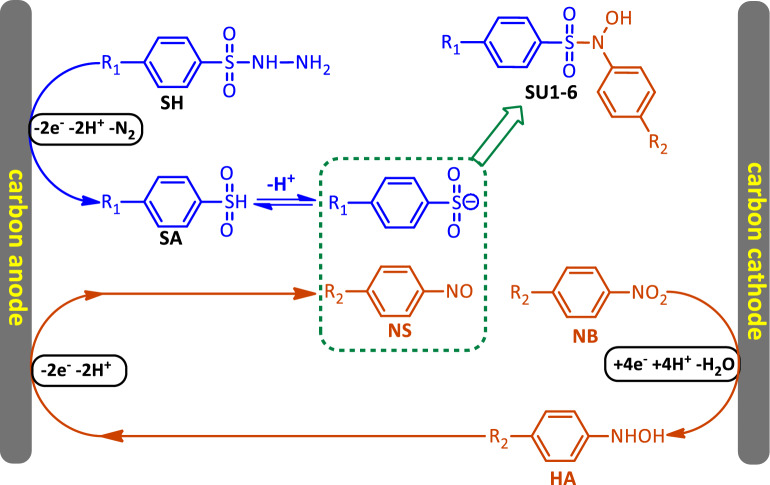
Proposed mechanism for the electrochemical synthesis of sulfonamides **SU1-6**.

Based on the obtained electrochemical results as well as the identification of the structure of the final products obtained from the electrolysis of **NB** in the presence of **SH**, the following mechanism is proposed for the synthesis of sulfonamides **SU1-6** (Fig. 6). According to Fig. 6, in the first step, the NO_2_ group of **NB** is reduced at the cathode to the hydroxylamine group by taking four electrons. In the next step, the hydroxylamine compound (**HA**) is oxidized at the anode to form the corresponding nitroso compound (**NS**) by losing two electrons and two protons. We refer to these two stages as the “*electrophilic generation step*”. On the other hand, the sulfonyl hydrazide (**SH**) is oxidized at the anode by losing two electrons, two protons, and releasing a N_2_ molecule to the corresponding sulfinic acid (**SA**). We refer to this stage as the "*nucleophilic formation step*". In the final stage, the produced nucleophile reacts with the generated electrophile to produce the final product (**SU1**-**6**) (Fig. 3, part III).

**Figure 7:**
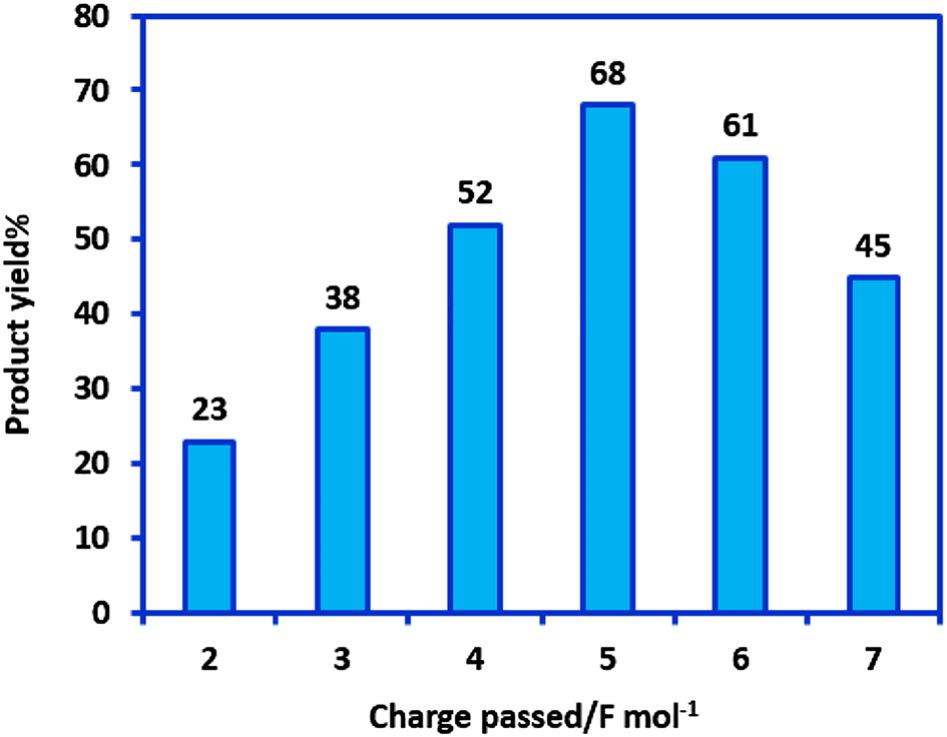
Effect of the charge passed on the yield of **SU2**. **NB2** amount: 0.5 mmol, **SH2** amount: 0.5 mmol, solvent: water (0.2 M phosphate buffer, pH = 2.0)/acetonitrile (50/50 v/v) mixture, applied potential: − 0.7 V versus Ag/AgCl. Anode: carbon and cathode: carbon. All experiments are performed at room temperature. These figures were prepared by Microsoft Excel (OFFICE 2013).

To improve the yield and purity of the synthesized sulfonamides, the amount of charge passing through the electrolytic cell was optimized. The condition of the experiment are as follows: applied potential: − 0.7 V versus Ag/AgCl, **NB2** amount: 0.5 mmol, **SH2** amount: 0.5 mmol, solvent: water (0.2 M phosphate buffer, pH = 2.0)/acetonitrile (50/50 v/v) mixture, anode: carbon and cathode: carbon. The results have been shown in Fig. 7. As can be seen, the product yield increases as the passing charge increases in the range of 2–5 F mol^−1^, but above this range, the yield of the product will be decreased. According to Fig. 6, the theoretical amount of electricity required for the synthesis of sulfonamides **SU1-6** is 4 F mol^−1^. Therefore, it can be expected that with the increase in the amount of electricity consumed, the yield will also increase, and reach the maximum. Nevertheless, since the current efficiency in this cell is not 100%, the maximum efficiency is obtained with the consumption of 5 F mol^−1^. The decrease in current efficiency from 100 is due to the necessity of using simple cell (undivided cell) and the occurrence of back reactions such as the reduction of **NS** in the cathode to **HA**. Based on the yield obtained at 4 F mol^−1^ and 5 F mol^−1^, the current efficiency and the amount of back reaction(s) in this cell can be considered equal to 52% and 23%, respectively. A further increase in electricity consumption causes a decrease in yield due to the occurrence of side reactions such as over-oxidation of the final product (**SU**).

Also, the effect of electrode type on the yield of the desired products was investigated. The results have been shown in Table 1 As can be seen, the highest yield is achieved when both anode and cathode are made of carbon.

**Table 1:** The effect of electrode type on the yield of **SU2**.


Entry	Cathode	Anode	Yield [%]
1	carbon	carbon	68
2	carbon	stainless steel	56
3	Fe	carbon	35
4	carbon	Fe	NR
5	Cu	carbon	36
6	stainless steel	carbon	25

Reaction conditions: **NB2** amount: 0.5 mmol. **SH2** amount: 0.5 mmol. Solvent: water (phosphate buffer, pH = 2.0, *c* = 0.2 M)/acetonitrile (50/50 v/v) mixture. *E*_app_ = − 0.7 V versus Ag/AgCl.

Under optimized conditions, we investigated the scope of the reaction using arenesulfonyl hydrazides (**SH1** and **SH2**) and some nitroarenes (**NB1**-**4**) (Table 2). The results show that we have succeeded in devising an ingenious method for the synthesis of sulphonamides **SU1-6** in a one-pot reaction with an overall yield of 59–68%. No data have been reported so far for the electrochemically synthesis of these sulfonamides. The reaction is carried out under mild conditions without using oxidizing or agents or catalysts at room temperature and atmospheric pressure.

**Table 2:** Scope of the sulfonamide derivatives synthesis.


Entry	Product	R_1_	R_2_	Yield [%]
1	**SU1**	–CH_3_	–H	61
2	**SU2**	–CH_3_	–CH_3_	68
3	**SU3**	–CH_3_	–Cl	64
4	**SU4**	–H	–H	59
5	**SU5**	–H	–Cl	65
6	**SU6**	–H	–I	67

## Conclusions

For the first time, we reported the electrochemical synthesis of sulfonamide compounds, with a new ping-pong type reaction mechanism. In this method, the activation of nitroarene and arylsulfonyl hydrazide as starting materials was necessary. In the first stage, the nitro group at the cathode surface was reduced to the hydroxylamine group and then it is oxidized at the anode surface to the corresponding nitroso compound (**NS**). On the other hand, **SH** compounds are oxidized to the corresponding sulfinic compounds at the anode surface. The chemical reaction between the two electrochemically produced precursors (**SA** and **NS**) leads to the corresponding sulfonamide. This procedure has many advantages over other reported methods such as: (I) Use of nitro compounds instead of toxic amine compounds, (II) Using electrons as a cost-effective and green oxidant instead of using chemical oxidants, (III) No need to use a catalyst, (IV) High current efficiency and low energy consumption. In convergent strategy, under ideal conditions, a current efficiency of 200% is achievable. Although our experiments were performed on a relatively small scale, there is little difficulty in producing larger quantities either by using larger cells or by running several cells in series.

### Supplementary Information

Supplementary Information.

## Data Availability

All data generated or analyzed during this study are included in this published article and its supplementary information file.

## References

[1] Buathongjan C, Beukeaw D, Yotphan S (2015). Iodine-catalyzed oxidative amination of sodium sulfinates: A convenient approach to the synthesis of sulfonamides under mild conditions. Eur. J. Org. Chem..

[2] Almalki AJ, Ibrahim TS, Taher ES, Mohamed MF, Youns M, Hegazy WA, Al-Mahmoudy AM (2022). Synthesis, antimicrobial, anti-virulence and anticancer evaluation of new 5(4*H*)-oxazolone-based sulfonamides. Molecules.

[3] Durgun M, Turkmen H, Zengin G, Zengin H, Koyunsever M, Koyuncu I (2017). Synthesis, characterization, in vitro cytotoxicity and antimicrobial investigation and evaluation of physicochemical properties of novel 4-(2-methylacetamide) benzenesulfonamide derivatives. Bioorg. Chem..

[4] Supuran CT, Casini A, Scozzafava A (2003). Protease inhibitors of the sulfonamide type: Anticancer, antiinflammatory, and antiviral agents. Med. Res. Rev..

[5] Alaoui S (2017). Synthesis and anti-cancer activities of new sulfonamides 4-substituted-triazolyl nucleosides. Bioorg. Med. Chem. Lett..

[6] Dai H-X, Stepan AF, Plummer MS, Zhang Y-H, Yu J-Q (2011). Divergent C-H functionalizations directed by sulfonamide pharmacophores: Late-stage diversification as a tool for drug discovery. J. Am. Chem. Soc..

[7] Lal J, Gupta SK, Thavaselvam D, Agarwal DD (2013). Biological activity, design, synthesis and structure activity relationship of some novel derivatives of curcumin containing sulphonamides. Eur. J. Med. Chem..

[8] Qin HL, Zhang Z-W, Lekkala R, Alsulami H, Rakesh K (2020). Chalcone hybrids as privileged scaffolds in antimalarial drug discovery: A key review. Eur. J. Med. Chem..

[9] Sayed AZ, El-Gaby MS (2001). Synthesis of novel dyestuffs containing sulphonamido moieties and their application on wool and polyamide fibres. Color. Technol..

[10] Jamshidi M, Amani A, Khazalpour S, Torabi S, Nematollahi D (2021). Progress and perspectives of electrochemical insights for C–H and N–H sulfonylation. New J. Chem..

[11] Tang X (2013). Copper-catalyzed sulfonamides formation from sodium sulfinates and amines. Chem. Commun..

[12] Yotphan S (2016). Iodine-catalyzed expeditious synthesis of sulfonamides from sulfonyl hydrazides and amines. Org. Biomol. Chem..

[13] Ji J, Liu Z, Liu P, Sun P (2016). Synthesis of sulfonamides via copper-catalyzed oxidative C–N bond cleavage of tertiary amines. Org. Biomol. Chem..

[14] Wei W (2015). Metal-free direct construction of sulfonamides via iodine- mediated coupling reaction of sodium sulfinates and amines at room temperature. Adv. Synth. Catal..

[15] Yu H, Zhang Y (2016). NH_4_I-catalyzed synthesis of sulfonamides from arylsufonylhydrazides and amines. Chin. J. Chem..

[16] Parumala SKR, Peddinti RK (2016). Metal-free synthesis of sulfonamides via iodine-catalyzed oxidative coupling of sulfonyl hydrazides and amines. Tetrahedron Lett..

[17] Yang K, Ke M, Lin Y, Song Q (2015). Sulfonamide formation from sodium sulfinates and amines or ammonia under metal-free conditions at ambient temperature. Green Chem..

[18] Yuan Y (2020). Independent phase modulation for quadruplex polarization channels enabled by chirality-assisted geometric-phase metasurfaces. Nat. Commun..

[19] Pandit SS, Pandit VU, Bandgar BP (2008). Rapid and efficient synthesis of sulfonamides from sulfonic acid and amines using cyanuric chloride-DMF adduct. J. Sulphur Chem..

[20] Pollok D, Waldvogel SR (2020). Electro-organic synthesis: A 21st century technique. Chem. Sci..

[21] Sadatnabi A, Mohamadighader N, Nematollahi D (2021). Convergent paired electrochemical synthesis of azoxy and azo compounds: An insight into the reaction mechanism. Org. Lett..

[22] Goljani H, Tavakkoli Z, Sadatnabi A, Masoudi-Khoram M, Nematollahi D (2020). A new electrochemical strategy for the synthesis of a new type of sulfonamide derivatives. Sci. Rep..

[23] Sadatnabi A, Nematollahi D (2022). An eco-friendly strategy using a double-current two-phase cell system for electrografting of polyacrylic acid. J. Electroanal. Chem..

[24] Aslam S, Sbei N, Rani S, Saad M, Fatima A, Ahmed N (2023). Heterocyclic electrochemistry: Renewable electricity in the construction of heterocycles. ACS Omega.

[25] Sbei N, Hardwick T, Ahmed N (2021). Electrochemical organic transformations via paired electrolysis. ACS Sustain. Chem. Eng..

[26] Chung S, Kim J (2019). Cu-catalyzed aerobic oxidative synthesis of sulfonamides from sulfonyl hydrazides and amines. Tetrahedron Lett..

[27] Chen J (2019). Iodine-catalyzed sulfonylation of sulfonyl hydrazides with tert-amines: A green and efficient protocol for the synthesis of sulphonamides. RSC Adv..

[28] Sheykhan M (2017). An approach to C–N activation: Coupling of arenesulfonyl hydrazides and arenesulfonyl chlorides with *tert*-amines via a metal-, oxidant- and halogen-free anodic oxidation. Green Chem..

[29] Poste AE, Grung M, Wright RF (2014). Amines and amine-related compounds in surface waters: A review of sources, concentrations and aquatic toxicity. Sci. Total Environ..

[30] Mokhtari B, Nematollahi D, Salehzadeh H (2018). Paired electrochemical conversion of nitroarenes to sulfonamides, diarylsulfones and bis (arylsulfonyl) aminophenols. Green Chem..

[31] Mokhtari B, Nematollahi D, Salehzadeh H (2019). A tunable pair electrochemical strategy for the synthesis of new benzenesulfonamide derivatives. Sci. Rep..

[32] Hauk A, Richartz H, Schramm KW, Fiedler H (1990). Reduction of nitrated phenols: A method to predict half-wave-potentials of nitrated phenols with molecular modelling. Chemosphere.

[33] Sandmeyer T (1884). Ueber die Ersetzung der Amid-gruppe durch Chlor, Brom und Cyan in den aromatischen Substanzen. Ber. Dtsch. Chem. Ges..

[34] Goljani H, Tavakkoli Z, Sadatnabi A, Nematollahi D (2020). Two-phase electrochemical generation of aryldiazonium salts: Application in electrogenerated copper-catalyzed sandmeyer reactions. Org. Lett..

[35] Alford E, Schofield K (1953). Cinnolines. Part XXX. The nature of the C (3)-position. The synthesis of 3-methyl-, 3-chloro-, and 3-bromo-cinnoline; also, of cinnoline and the Bz-nitrocinnolines. J. Chem. Soc. (Resumed).

[36] Li W (2017). Catalyst-free synthesis of 3-sulfone nitrile from sulfonyl hydrazides and acrylonitrile in water. Org. Biomol. Chem..

