# Supplementary material for: A new type of convergent paired electrochemical synthesis of sulfonamides under green and catalyst-free conditions
Source: Sci Rep. 2023 Oct 16;13:17582. doi: 10.1038/s41598-023-44912-y (PMC10579442; doi:10.1038/s41598-023-44912-y)

Supplementary Information

A new type of convergent paired electrochemical synthesis of sulfonamides under green and catalyst-free conditions. An example of oxidative nucleophile generation

Pouria Patoghi, Ali Sadatnabi, Davood Nematollahi*

Faculty of Chemistry, Bu-Ali Sina University, Hamedan, Iran. Zip Code 65178-38683. Corresponding author. Tel.: + 0098 813 8271541; fax: +0098 813 8272404. E-mail addresses: nemat@basu.ac.ir, dnematollahi@yahoo.com (D. Nematollahi).

Table of Contents:

FT-IR spectrum of **SU1**.. Page S3

^{1}H NMR spectrum of **SU1**.. Page S4

Expanded ^{1}H NMR spectrum of **SU1**.. Page S5

FT-IR spectrum of **SU2**.. Page S6

^{1}H NMR spectrum of **SU2**.. Page S7

Expanded ^{1}H NMR spectrum of **SU2**.. Page S8

Mass spectrum of **SU2**.. Page S9

FT-IR spectrum of **SU3**.. Page S10

^{1}H NMR spectrum of **SU3**.. Page S11

Expanded ^{1}H NMR spectrum of **SU3**.. Page S12

FT-IR spectrum of **SU4**.. Page S13

^{1}H NMR spectrum of **SU4**.. Page S14

Expanded ^{1}H NMR spectrum of **SU4**.. Page S15

FT-IR spectrum of **SU5**.. Page S16

^{1}H NMR spectrum of **SU5**.. Page S17

Expanded ^{1}H NMR spectrum of **SU5**.. Page S18

FT-IR spectrum of **SU6**.. Page S19

^{1}H NMR spectrum of **SU6**.. Page S20

Expanded ^{1}H NMR spectrum of **SU6**.. Page S21

FT-IR spectrum of SU1

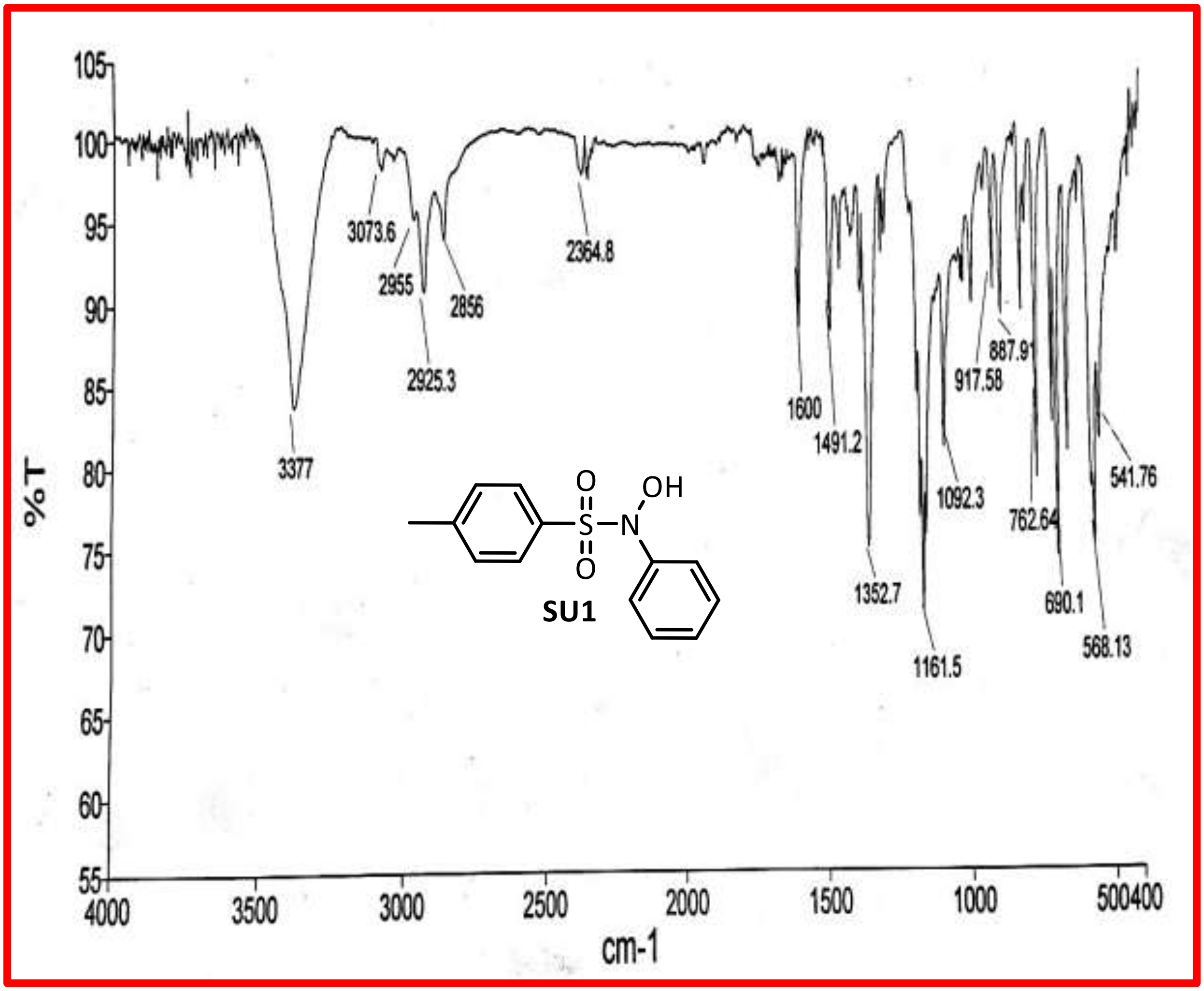

^{1}H NMR spectrum of SU1

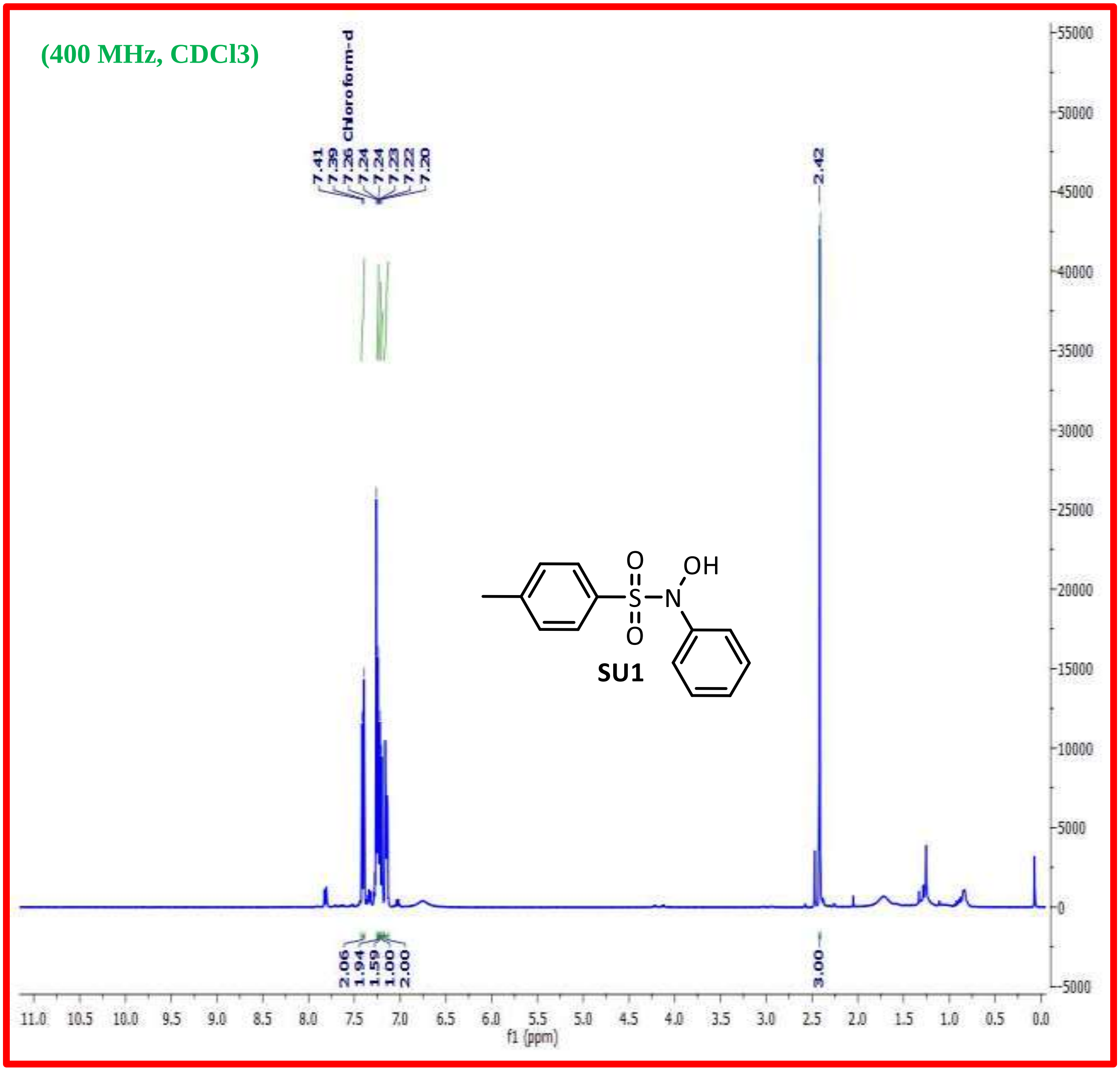

Expanded ^{1}H NMR spectrum of SU1

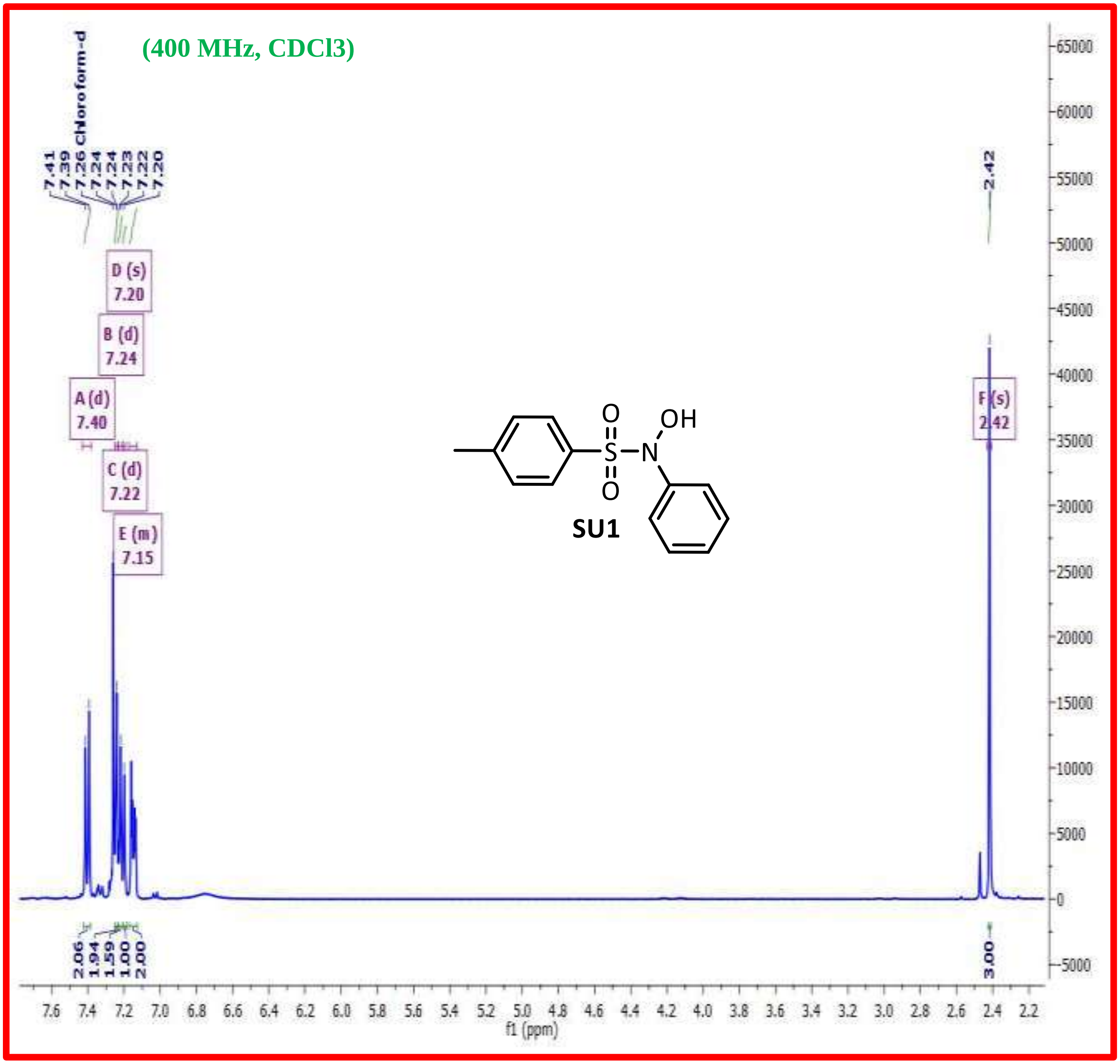

FT-IR spectrum of SU2

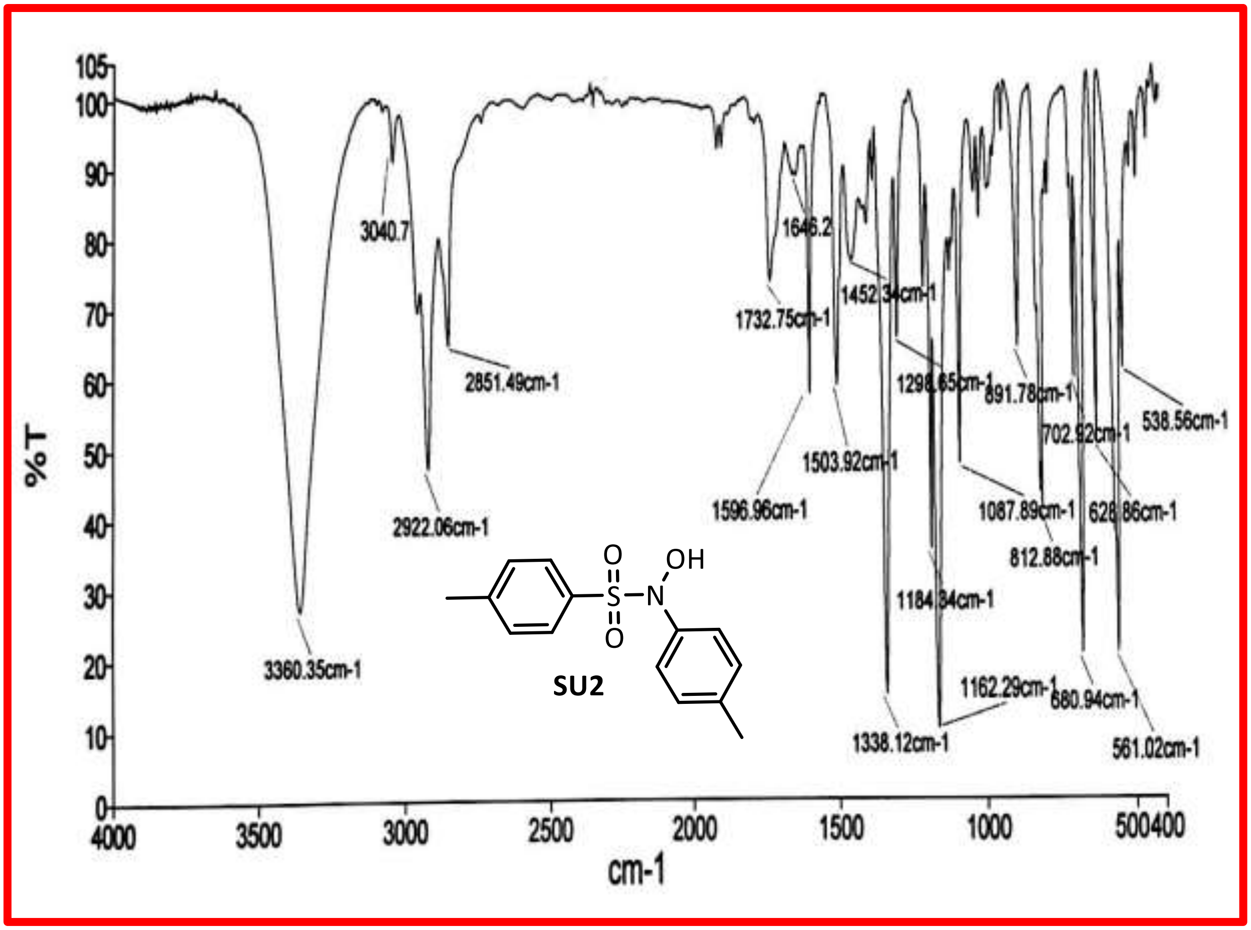

^{1}H NMR spectrum of SU2

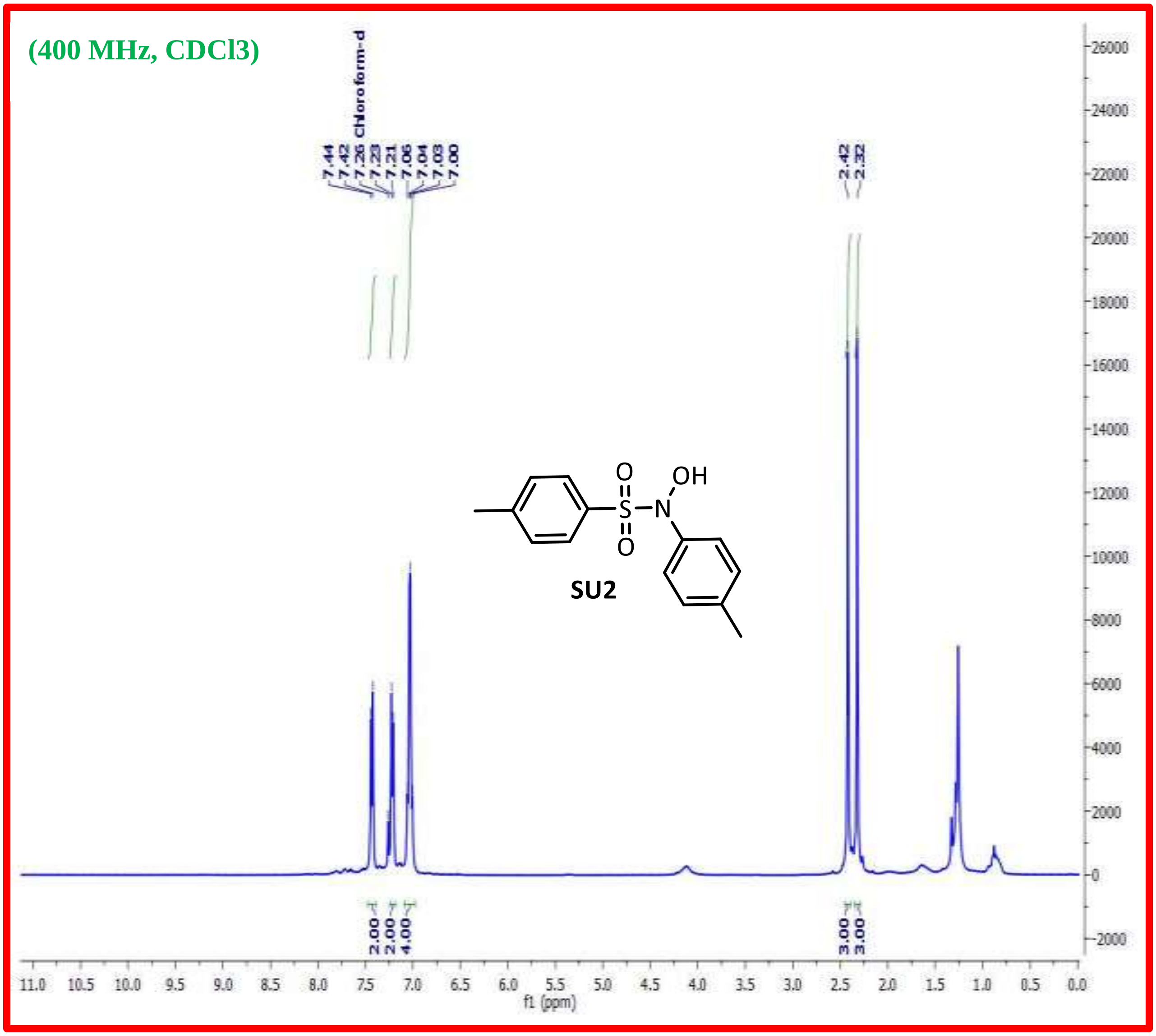

Expanded ^{1}H NMR spectrum of SU2

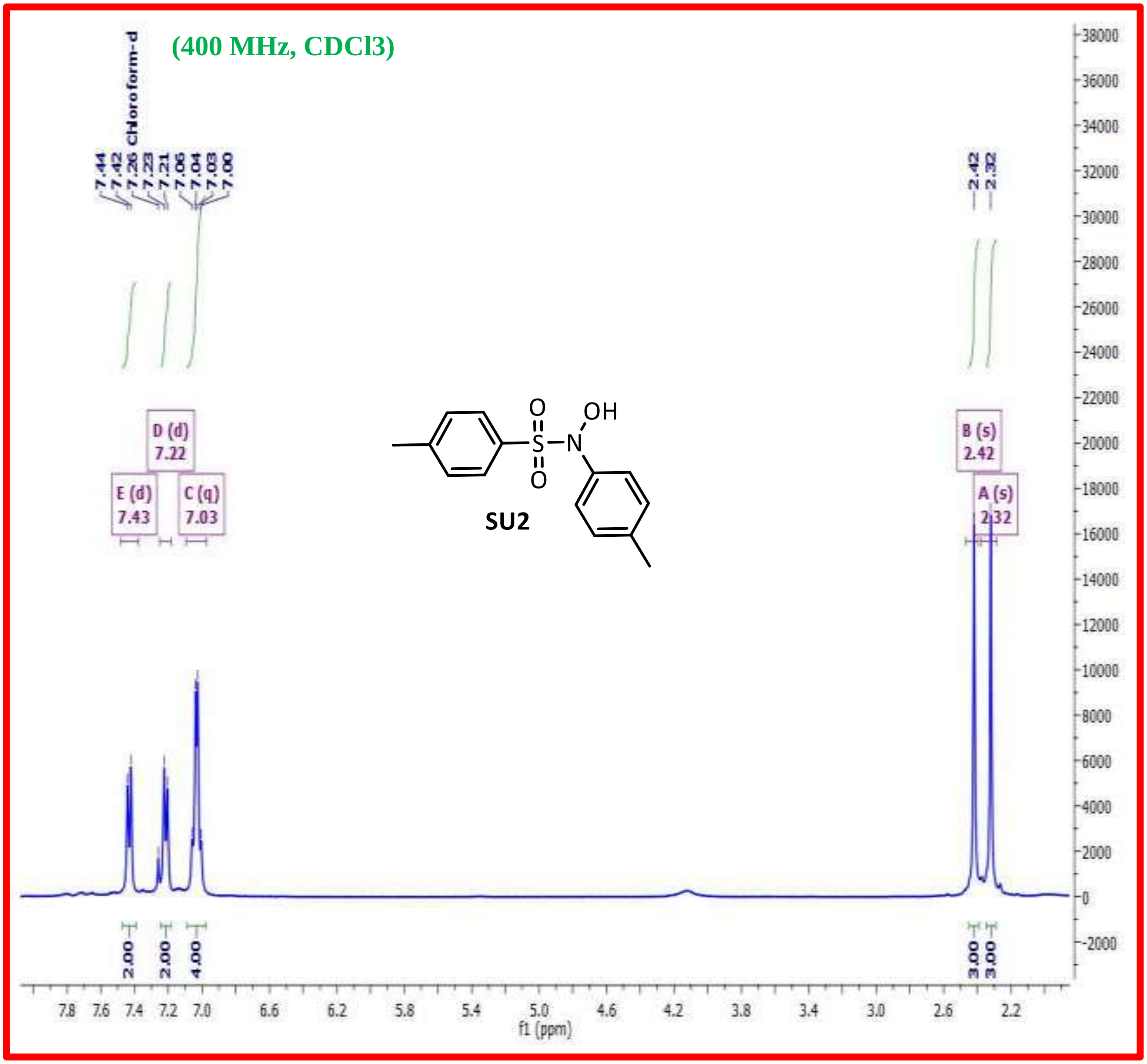

MS spectrum of SU2

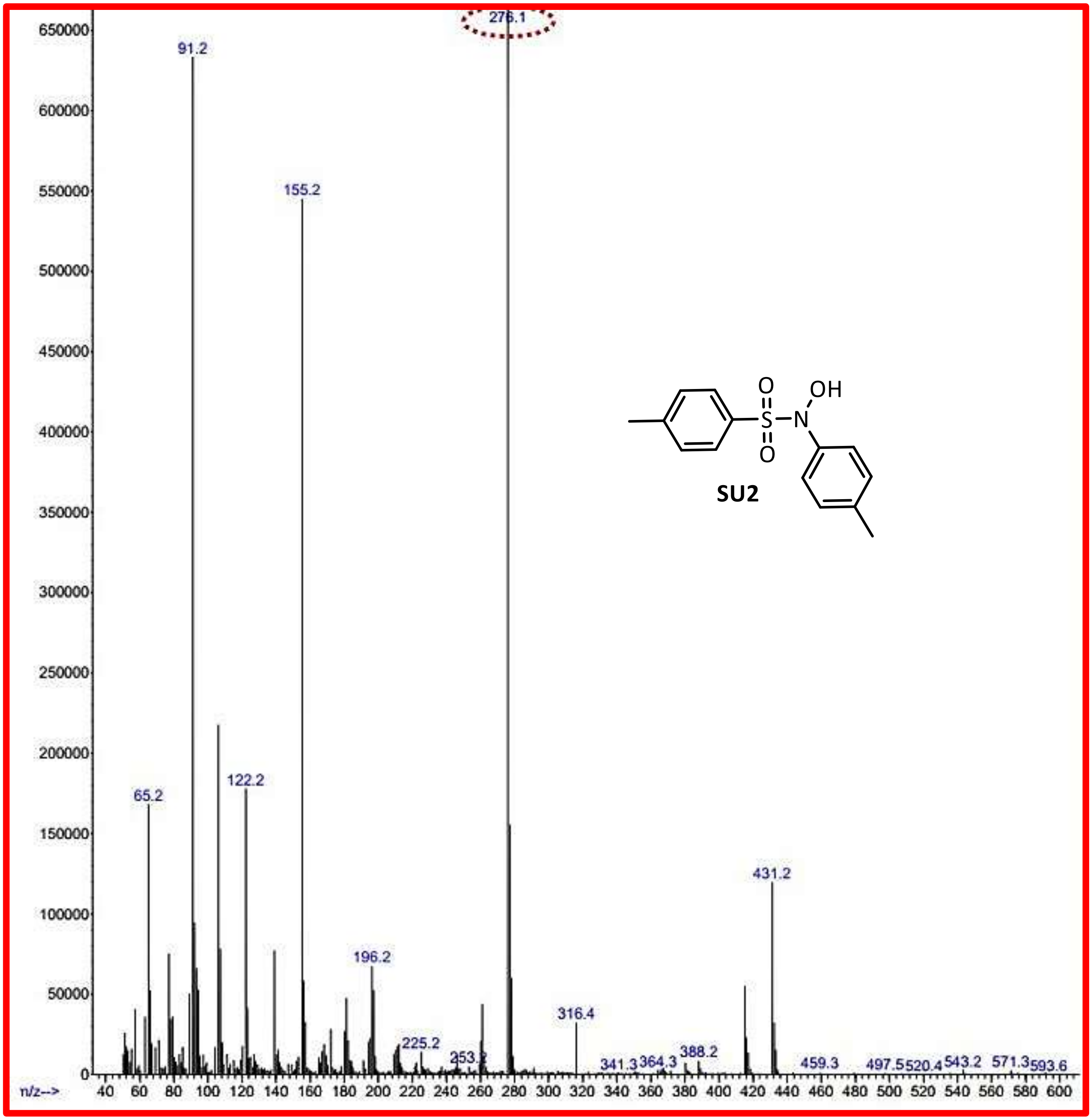

FT-IR spectrum of SU3

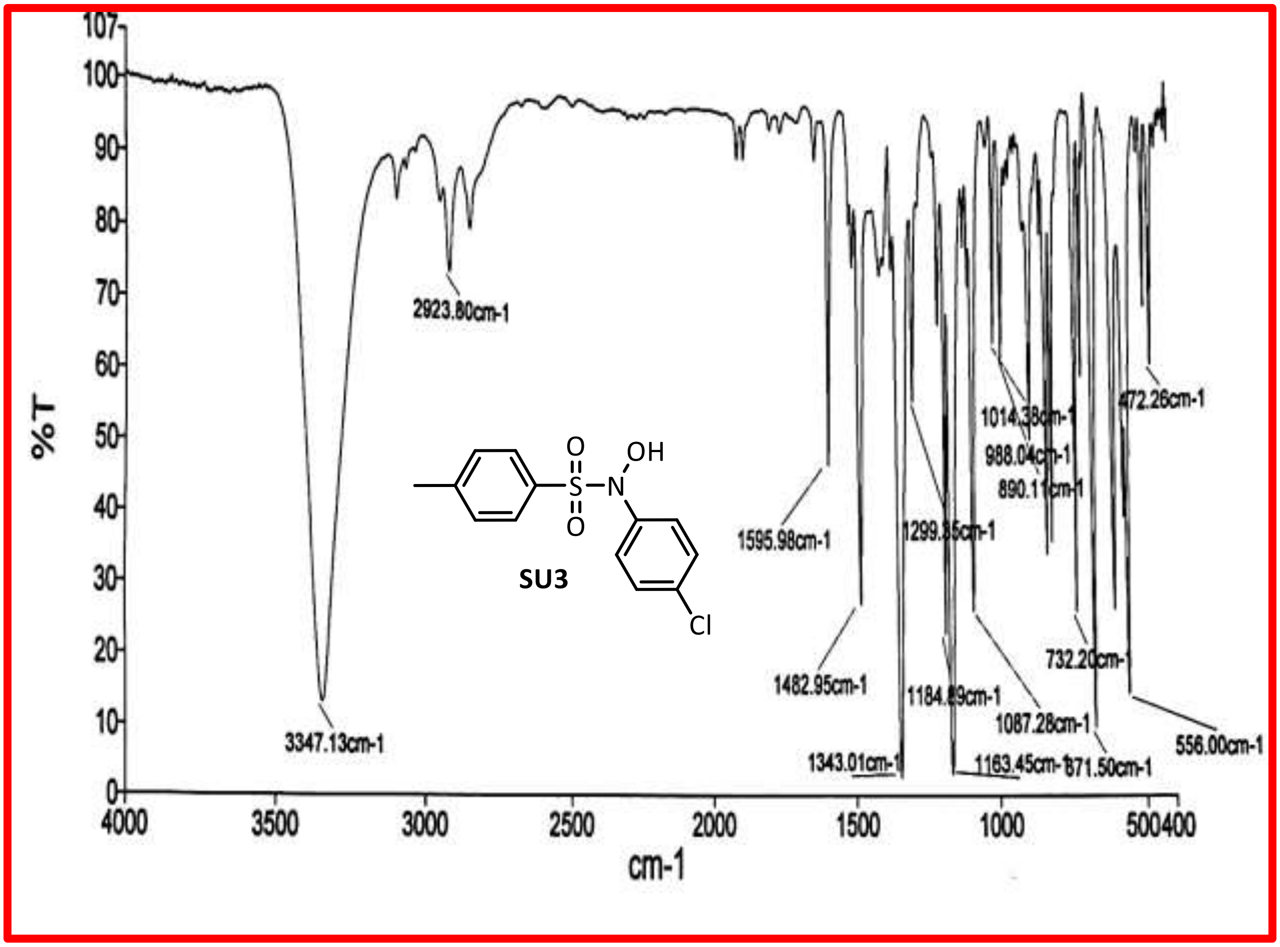

^{1}H NMR spectrum of SU3

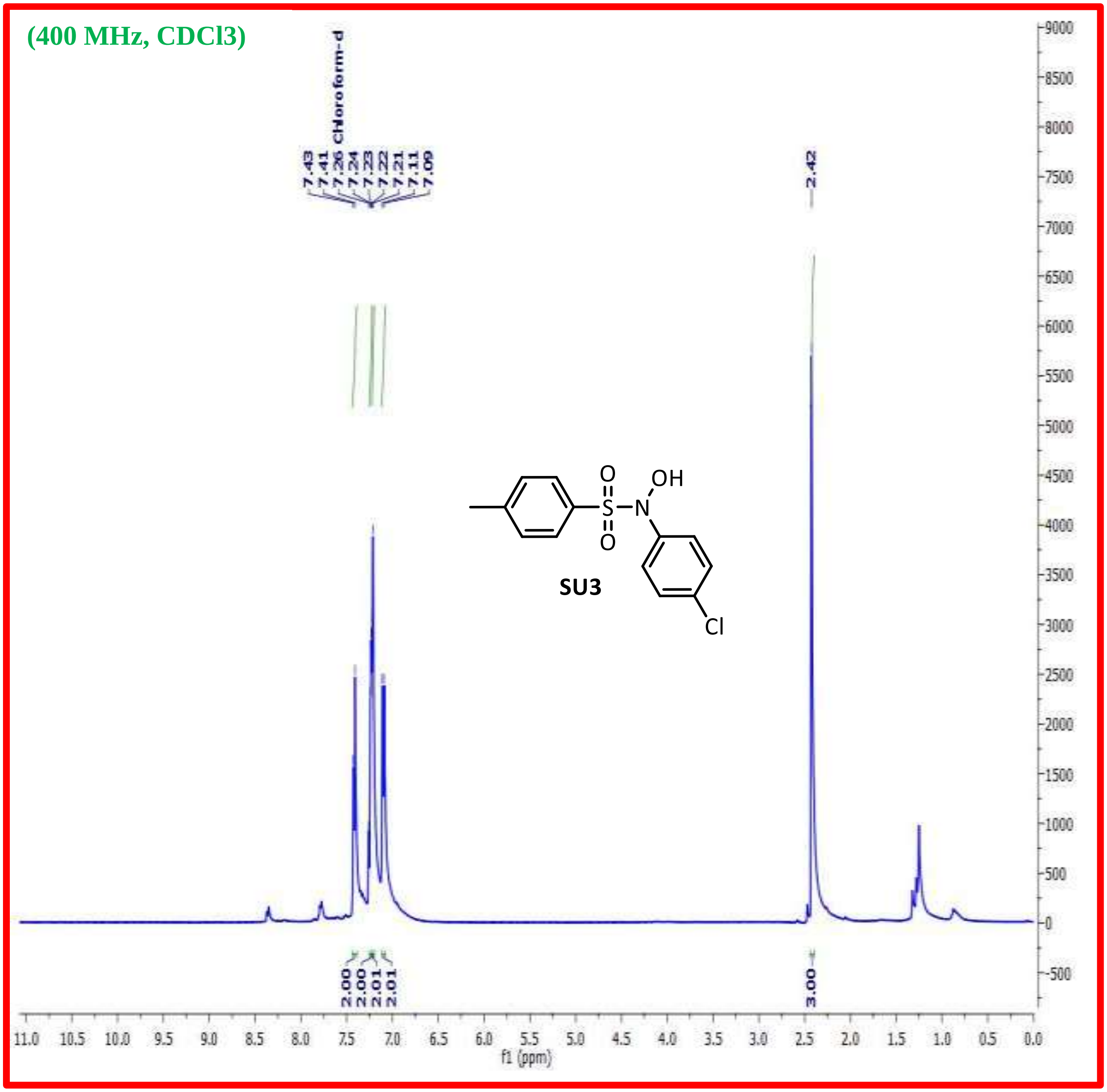

Expanded ^{1}H NMR spectrum of SU3

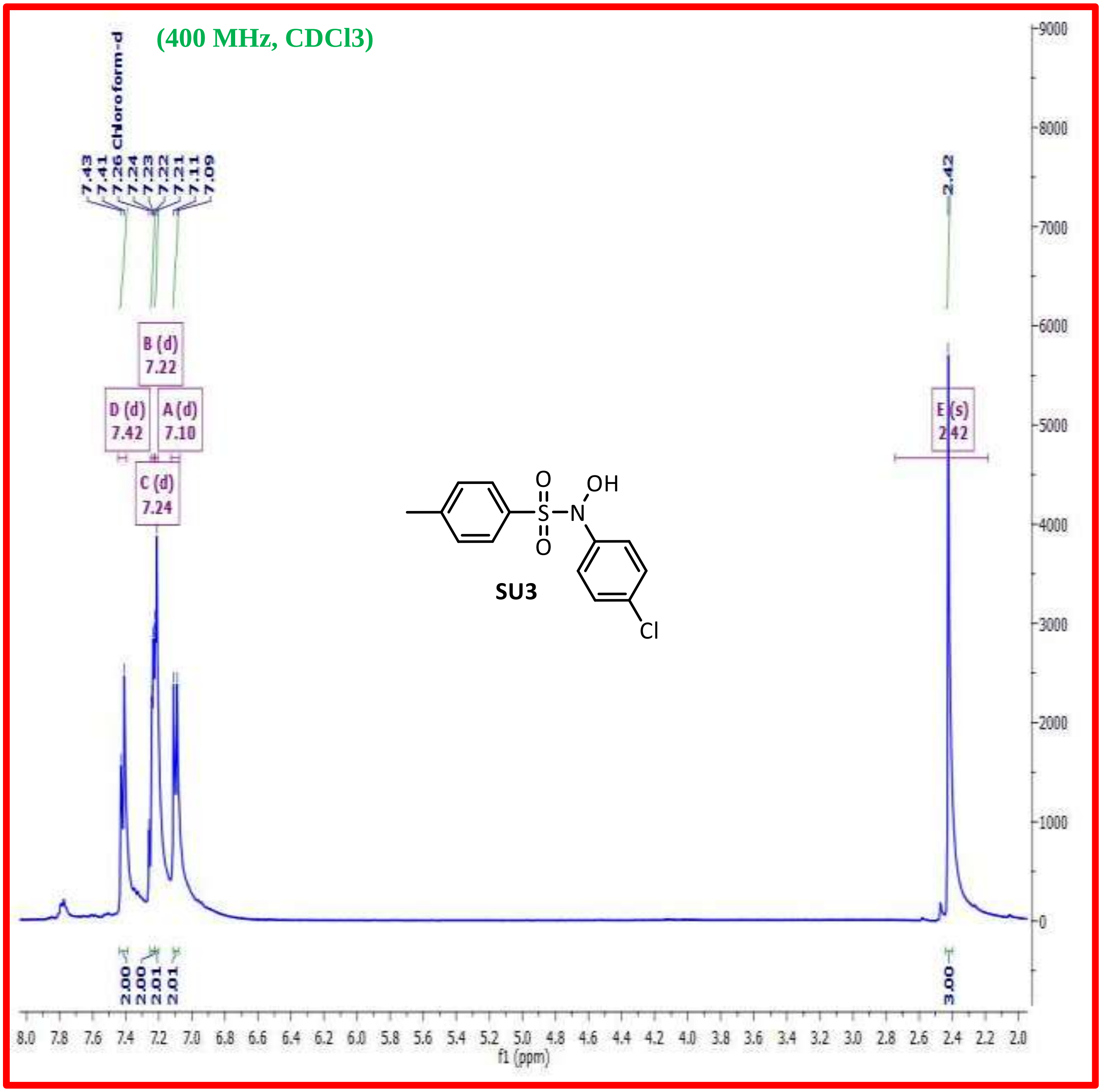

FT-IR spectrum of SU4

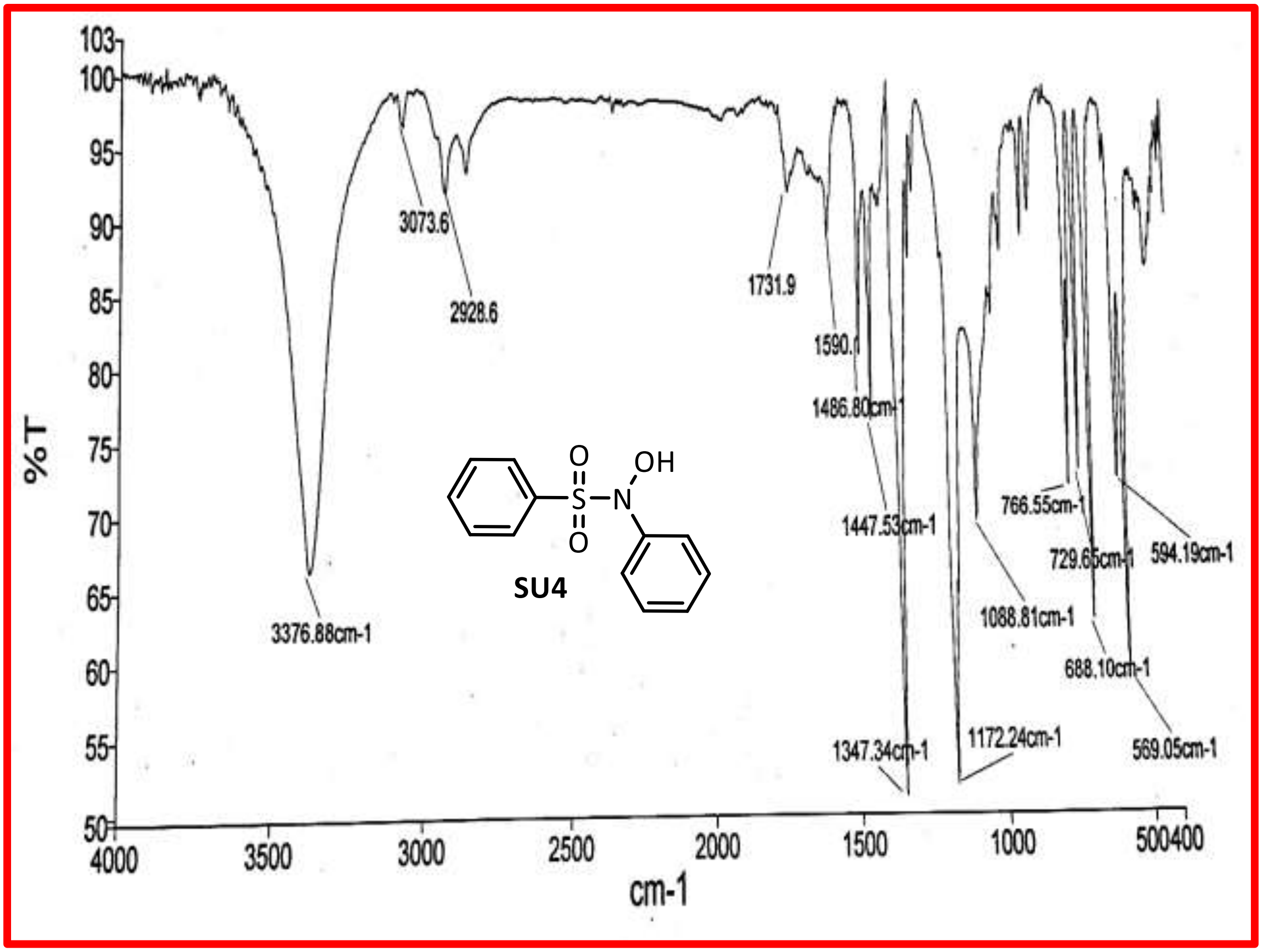

^{1}H NMR spectrum of SU4

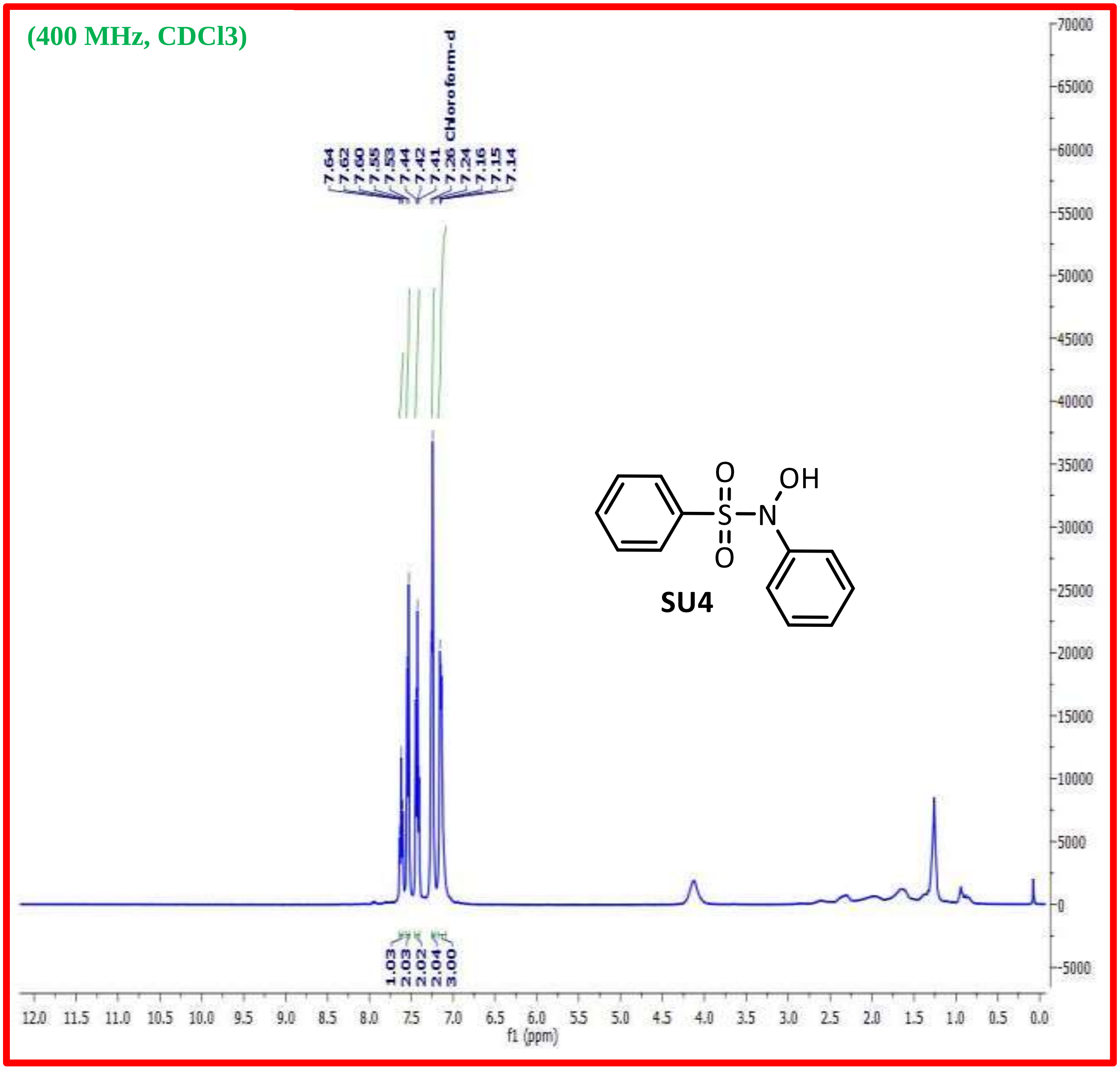

Expanded ^{1}H NMR spectrum of 1b

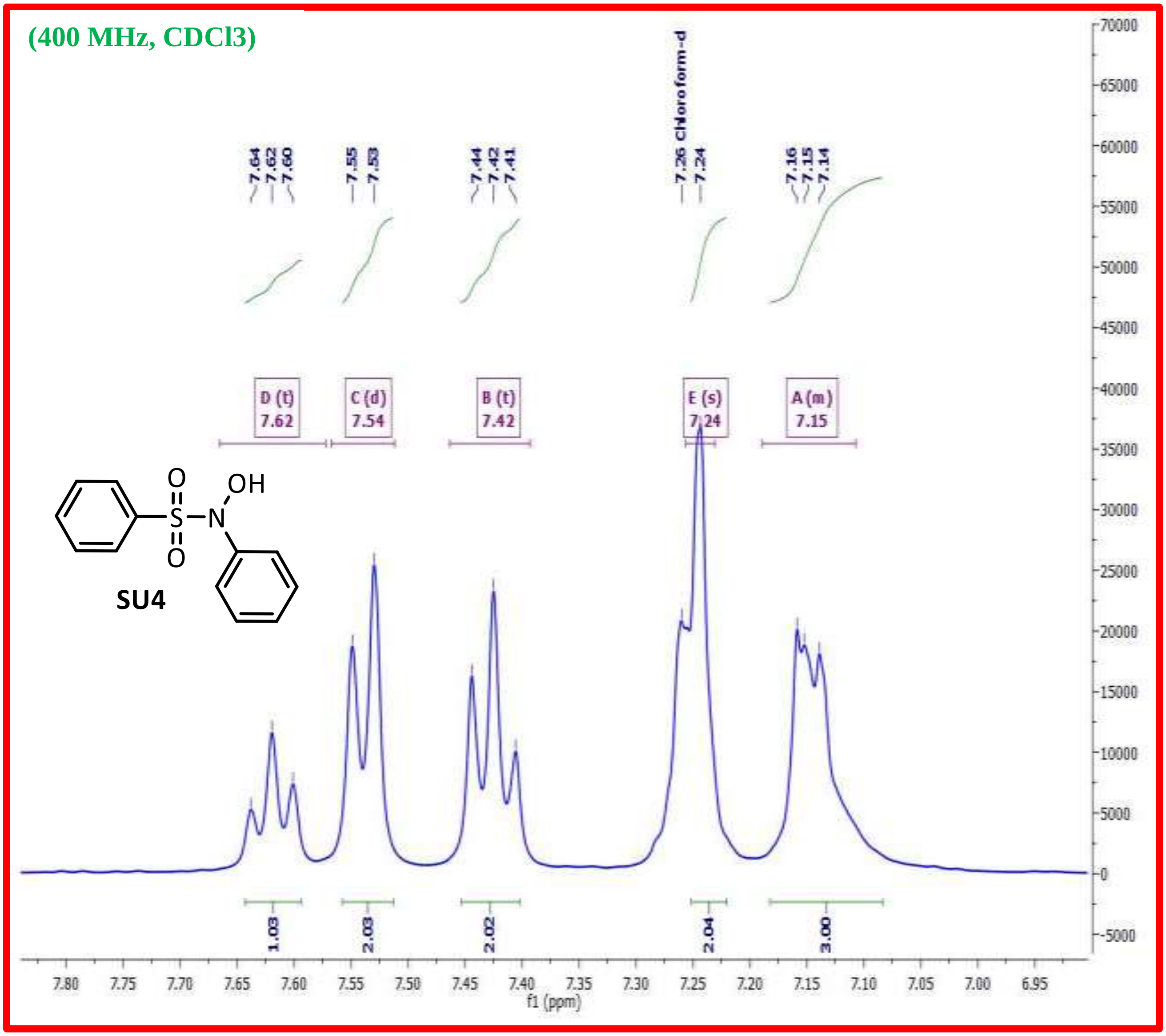

FT-IR spectrum of SU5

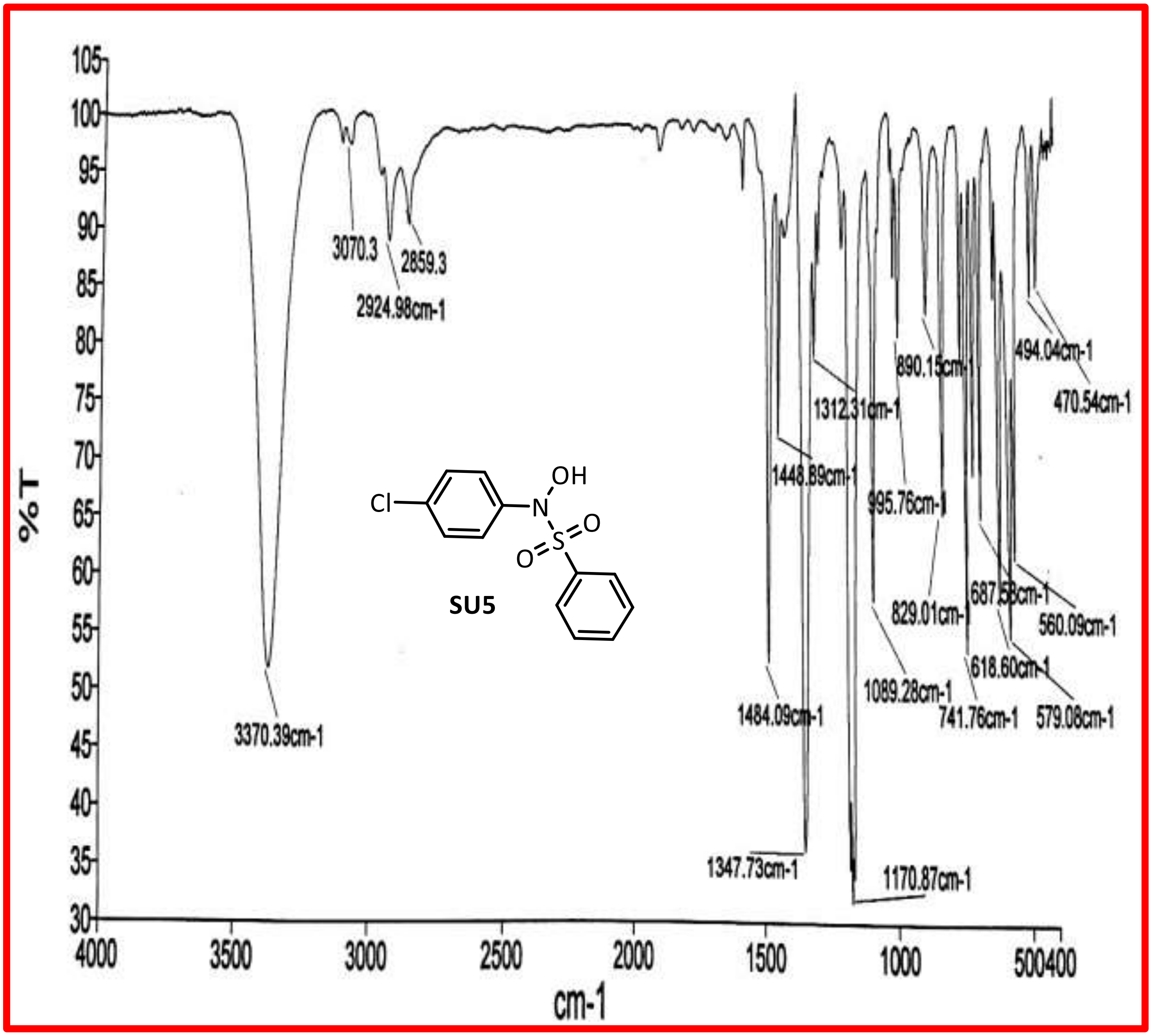

^{1}H NMR spectrum of SU5

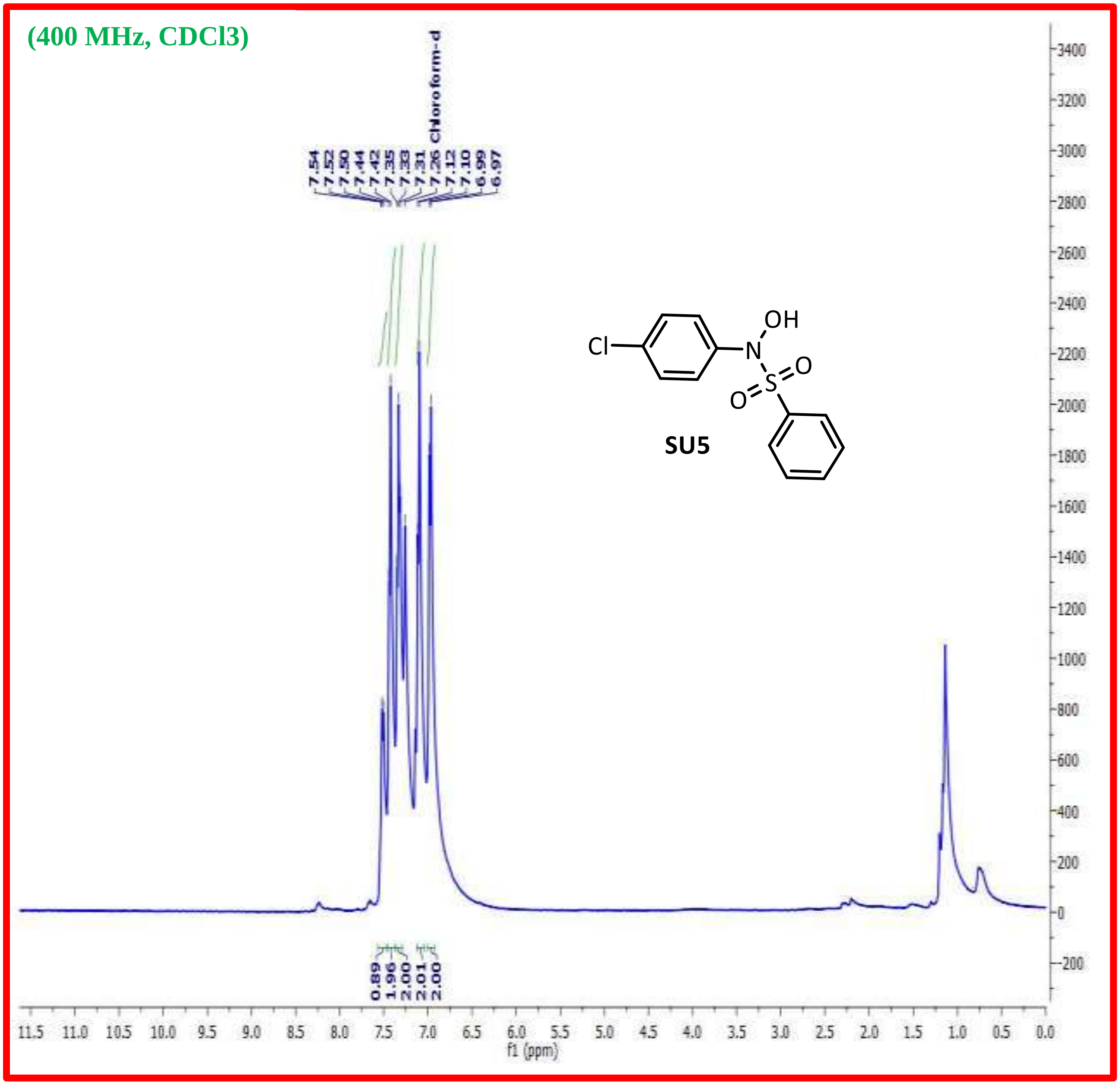

Expanded ^{1}H NMR spectrum of SU5

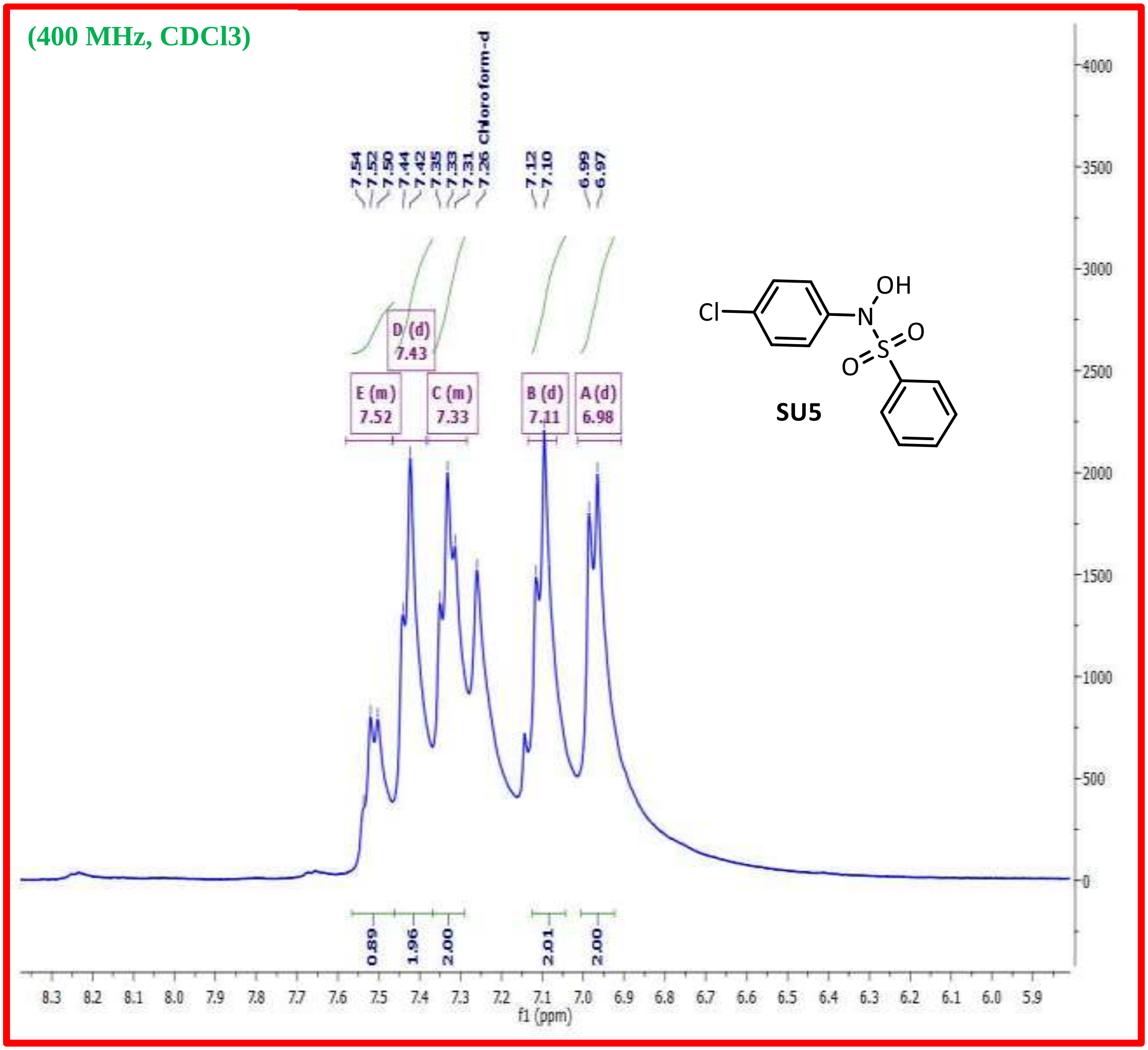

FT-IR spectrum of SU6

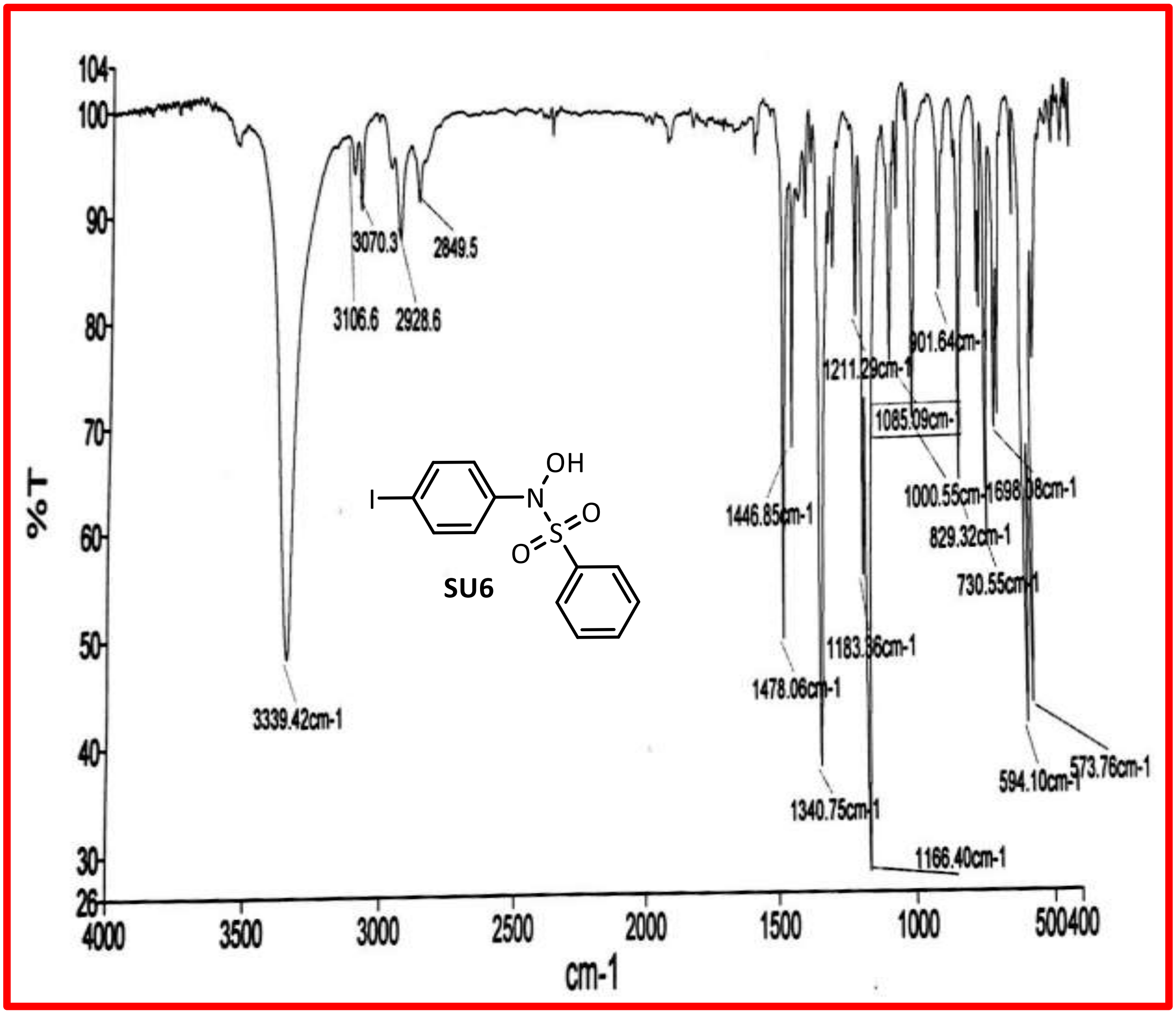

^{1}H NMR spectrum of SU6

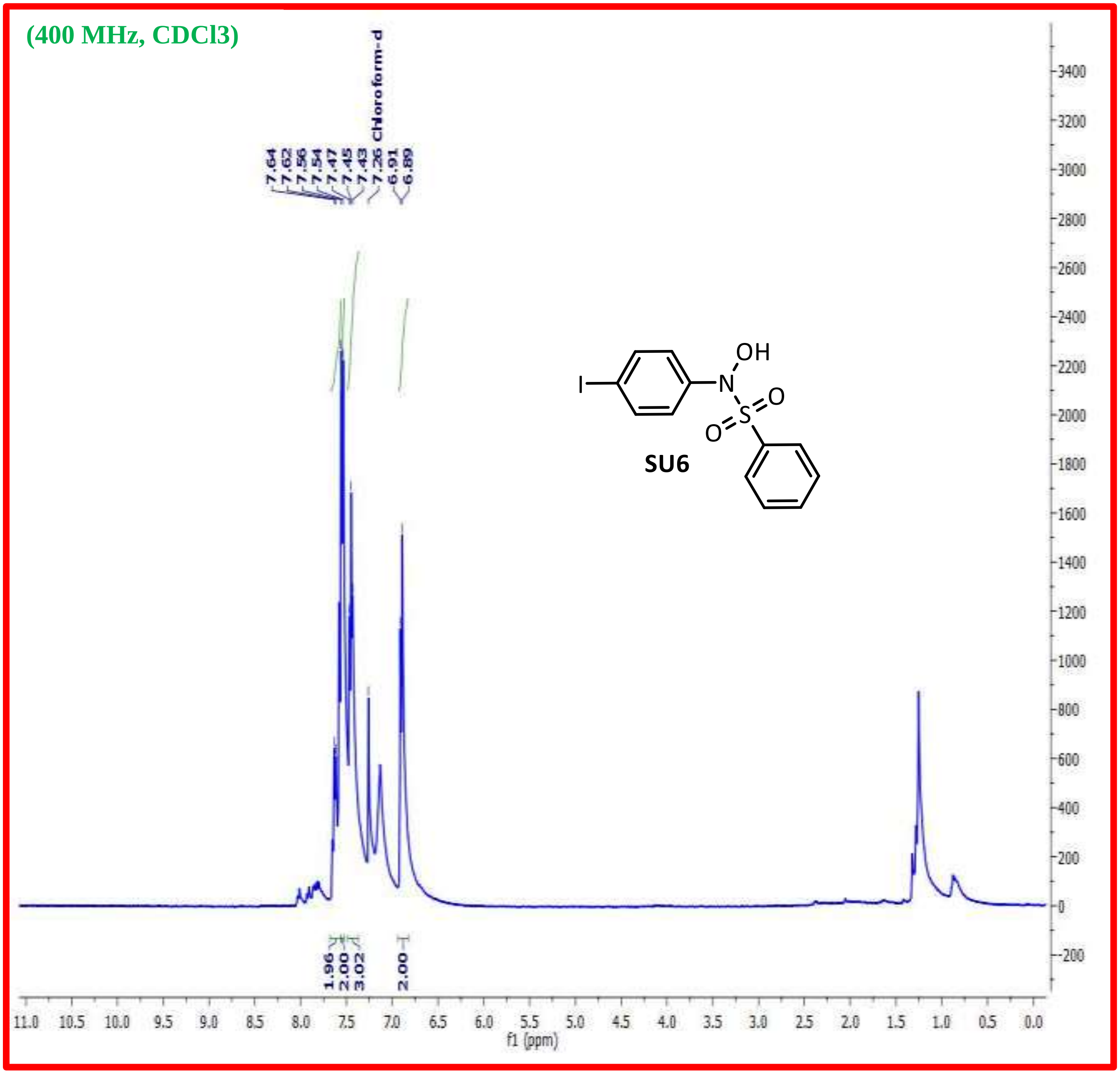

Expanded ^{1}H NMR spectrum of SU6

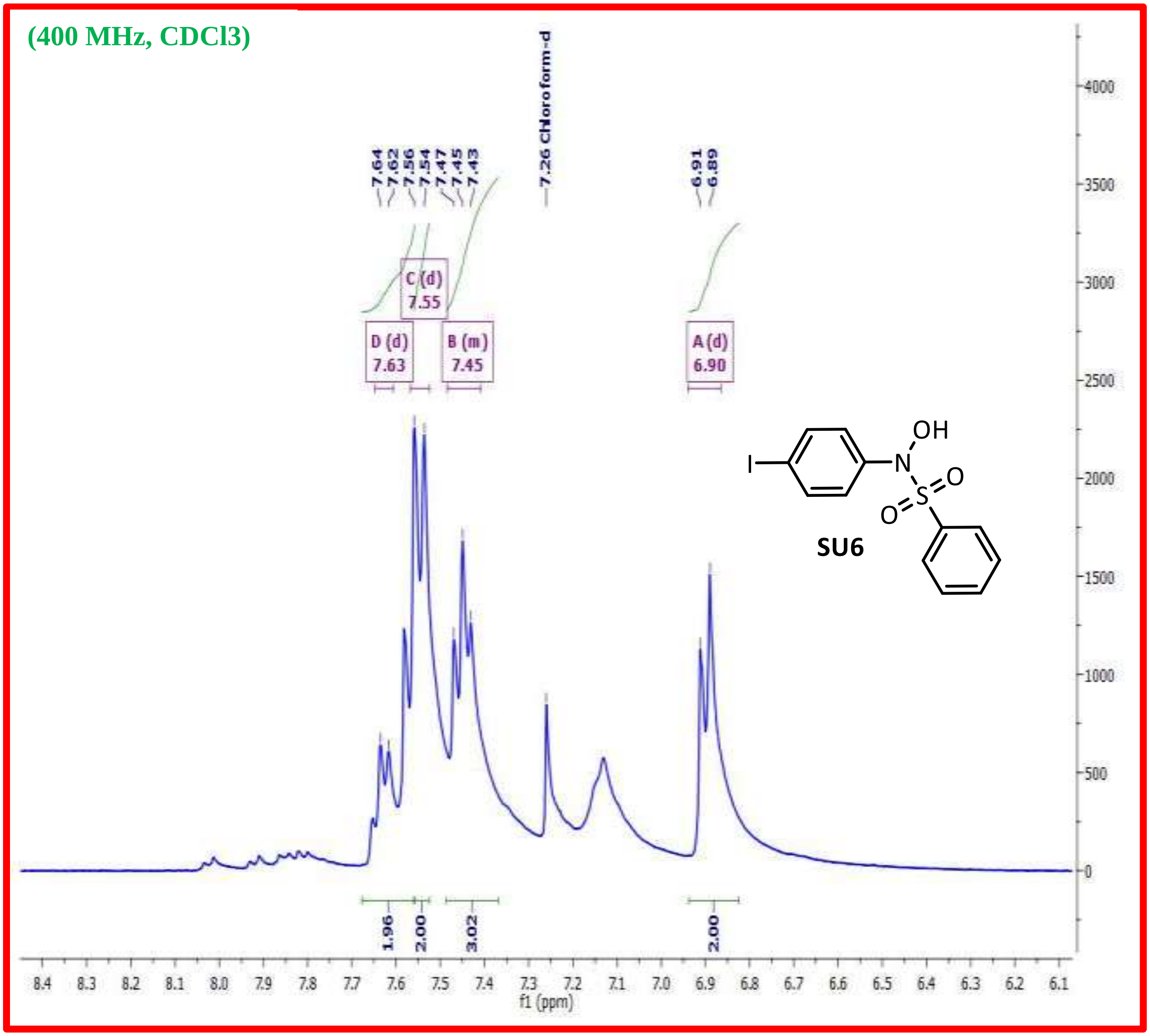

Supplement: Supplementary file 1 — Supplementary Information. [file 41598_2023_44912_MOESM1_ESM.pdf]
